# Thrombopoietin mimetic reduces mouse lung inflammation and fibrosis after radiation by attenuating activated endothelial phenotypes

**DOI:** 10.1172/jci.insight.181330

**Published:** 2024-11-08

**Authors:** Jeb English, Sriya Dhanikonda, Kathryn E. Tanaka, Wade Koba, Gary Eichenbaum, Weng-Lang Yang, Chandan Guha

**Affiliations:** 1 Department of Radiation Oncology,; 2 Department of Pathology, and; 3 Department of Radiology, Albert Einstein College of Medicine, Bronx, New York, USA.; 4 Office of the Chief Medical Officer, Johnson & Johnson, New Brunswick, New Jersey, USA.

**Keywords:** Pulmonology, Therapeutics, Endothelial cells, Fibrosis, Radiation therapy

## Abstract

Radiation-induced lung injury (RILI) initiates radiation pneumonitis and progresses to fibrosis as the main side effect experienced by patients with lung cancer treated with radiotherapy. There is no effective drug for RILI. Sustained vascular activation is a major contributor to the establishment of chronic disease. Here, using a whole thoracic irradiation (WTI) mouse model, we investigated the mechanisms and effectiveness of thrombopoietin mimetic (TPOm) for preventing RILI. We demonstrated that administering TPOm 24 hours before irradiation decreased histologic lung injury score, apoptosis, vascular permeability, expression of proinflammatory cytokines, and neutrophil infiltration in the lungs of mice 2 weeks after WTI. We described the expression of c-MPL, a TPO receptor, in mouse primary pulmonary microvascular endothelial cells, showing that TPOm reduced endothelial cell–neutrophil adhesion by inhibiting ICAM-1 expression. Seven months after WTI, TPOm-treated lung exhibited less collagen deposition and expression of MMP-9, TIMP-1, IL-6, TGF-β, and p21. Moreover, TPOm improved lung vascular structure, lung density, and respiration rate, leading to a prolonged survival time after WTI. Single-cell RNA sequencing analysis of lungs 2 weeks after WTI revealed that TPOm shifted populations of capillary endothelial cells toward a less activated and more homeostatic phenotype. Taken together, TPOm is protective for RILI by inhibiting endothelial cell activation.

## Abstract 2

TPOm reduces radiation-induced lung injury by targetting and modulating lung capillary endothelial cells

## Graphical abstract

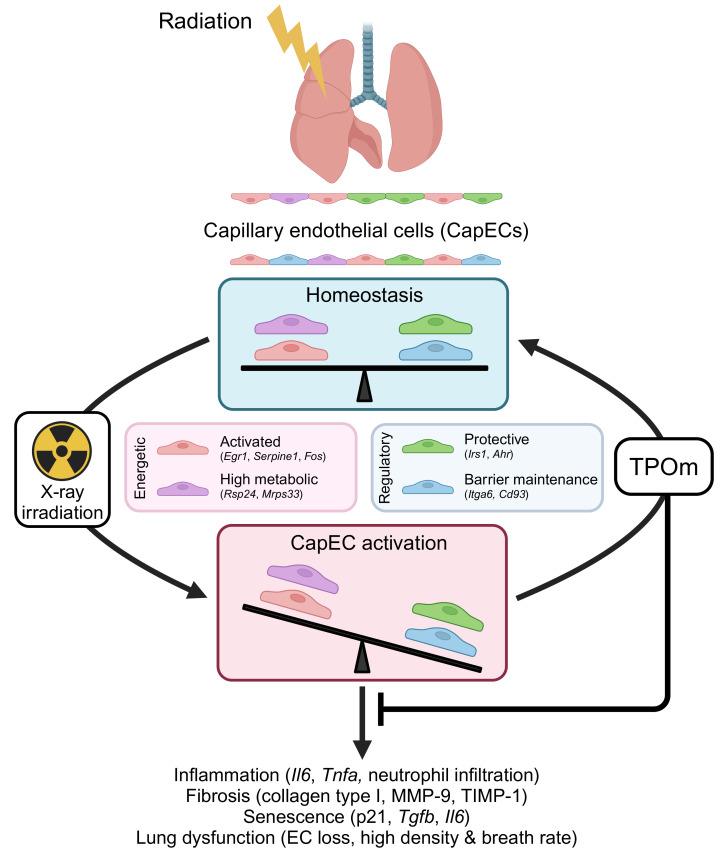

## Introduction

Radiotherapy is a key treatment modality for thoracic malignancies, often used in combination with chemotherapy and surgery to promote local tumor control (1). However, the secondary effects of radiotherapy on noncancerous tissue limit the radiation dose, resulting in risk of poor tumor control. Local high-dose radiation exposure to the lung causes radiation-induced lung injury (RILI), which presents with symptoms such as coughing, shortness of the breath, and chest pain, for months to years after exposure (2). In the population of 235,000 newly diagnosed patients with lung cancer per year in the US, roughly 17% of those who receive radiotherapy will experience pneumonitis and will be at high risk for fibrosis (3). Fibrosis leads to a decline in pulmonary function and an increasing likelihood of mortality (4). Corticosteroids are the standard of care for reducing inflammation in RILI (5). However, there are currently no approved therapies to mitigate radiation-induced pulmonary fibrosis for patients, and steroid administration has limited effectiveness once fibrosis is present.

Radiation pneumonitis occurring from RILI can last from weeks to months after the initial radiation insult and is characterized by inflammation (6). Radiation causes necrosis and apoptosis of epithelial cells and endothelial cells (ECs) in the alveoli early after exposure, which results in releasing damage-associated molecular patterns for stimulating inflammation (7). The elevation of proinflammatory cytokines, such as IL-6, and chemokines, such as monocyte chemoattractant protein 1 (MCP-1) and keratinocyte chemokine (KC), recruits immune effector cells like macrophages and neutrophils to assist in remodeling damaged tissues (8). High accumulation of recruited immune cells in the damaged tissues results in dysregulation of paracrine and autocrine signaling among macrophages, fibroblasts, epithelium, and endothelium (9). During RILI, ECs are increasingly activated to express cell adhesion molecules, such as intercellular cell adhesion molecule 1 (ICAM-1), which can attract the binding of neutrophils, subsequently degrading tissue using proteases (10). Furthermore, the degraded endothelium results in the loss of the endothelial barrier, which allows plasma proteins and excessive immune cell infiltration into alveolar space, which has been observed in acute respiratory distress syndrome (ARDS) (11). Disruption of the lung endothelial barrier has been considered a major cause of morbidity and mortality in acute lung injury and ARDS (12).

Unresolved vascular-mediated inflammation primes the lung for development of fibrosis months to years after radiation exposure, characterized by collagen deposition, fibroblast activation, and proliferation (13). Oxidative and DNA damage from radiation exposure, as well as the overwhelming microenvironmental factors released by other cell types, encourage alveolar cells to enter into a senescent state characterized by cell cycle arrest (14). Senescent cells will adopt a senescence-associated secretory phenotype (SASP), adding to the proinflammatory and profibrotic microenvironment in the lung (15). A key player in the profibrotic microenvironment is TGF-β, found to be expressed across models of chronic inflammation (16). The swath of paracrine factors sustains aberrant fibroblast activation, allowing for the excessive deposition of collagens type I and IV, matrix metalloproteases (MMPs), and tissue inhibitors of metalloproteinases (TIMPs) (17, 18). In tissue remodeling, the once functional endothelium is now replaced by fibrotic, collagen-dense lesions (2). The increased lung density from collagen deposition and the loss of vascularization results in a widening of the alveolar septa and less efficient gas exchange, resulting in an ultimate decline in pulmonary function (4).

Thrombopoietin mimetic (TPOm), or JNJ-26366821, is a synthetic, PEGylated peptide of 29 amino acids, which is discovered by phage display screening for binding to c-MPL, a TPO receptor (19), and has recently shown efficacy and safety as a radiomitigator in preclinical mouse models of acute radiation syndrome (20–22). c-MPL is expressed on ECs in multiple tissues (23, 24). ECs respond to TPO stimulation, inducing angiogenic expansion and activating prosurvival signaling pathways (25, 26). A recent study demonstrated that TPOm protected microvasculature against radiation-induced injury (27). Given the importance of endothelial dysfunction to the progression of RILI, it can be hypothesized that ECs could be protected by TPOm treatment to prevent downstream damage and tissue fibrosis after radiation exposure.

In this study, we used a whole thoracic irradiation (WTI) mouse model to evaluate the efficacy of TPOm in treating RILI. Studies were conducted in C57BL/6J mouse strain, which presents both lung inflammation and fibrosis after WTI (28). TPOm was administered one day prior to WTI. We first examined the radiation pneumonitis stage 2 weeks after irradiation (IR) by comparing the lung damage, apoptosis, leakage, inflammation, and neutrophil infiltration between vehicle- and TPOm-treated mice. We also isolated mouse pulmonary microvascular ECs (PMVECs) to study the effect of TPOm on regulating the activation of ECs induced by IR. We then examined the lung fibrosis stage 7 months after IR by comparing histology, collagen deposition, MMP expression, senescence, vascularity, lung density and function between vehicle- and TPOm-treated mice. We also examined the effect of TPOm treatment on the survival time of WTI mice. Finally, we performed single-cell RNA sequencing (scRNA-Seq) on nonhematopoietic lung cells from mice 2 weeks after WTI to further understand the effect of TPOm on changing the phenotypes of various cell types, especially on ECs.

## Results

### TPOm reduces lung injury, barrier dysfunction, and inflammation in mice after WTI.

**Figure 1:**
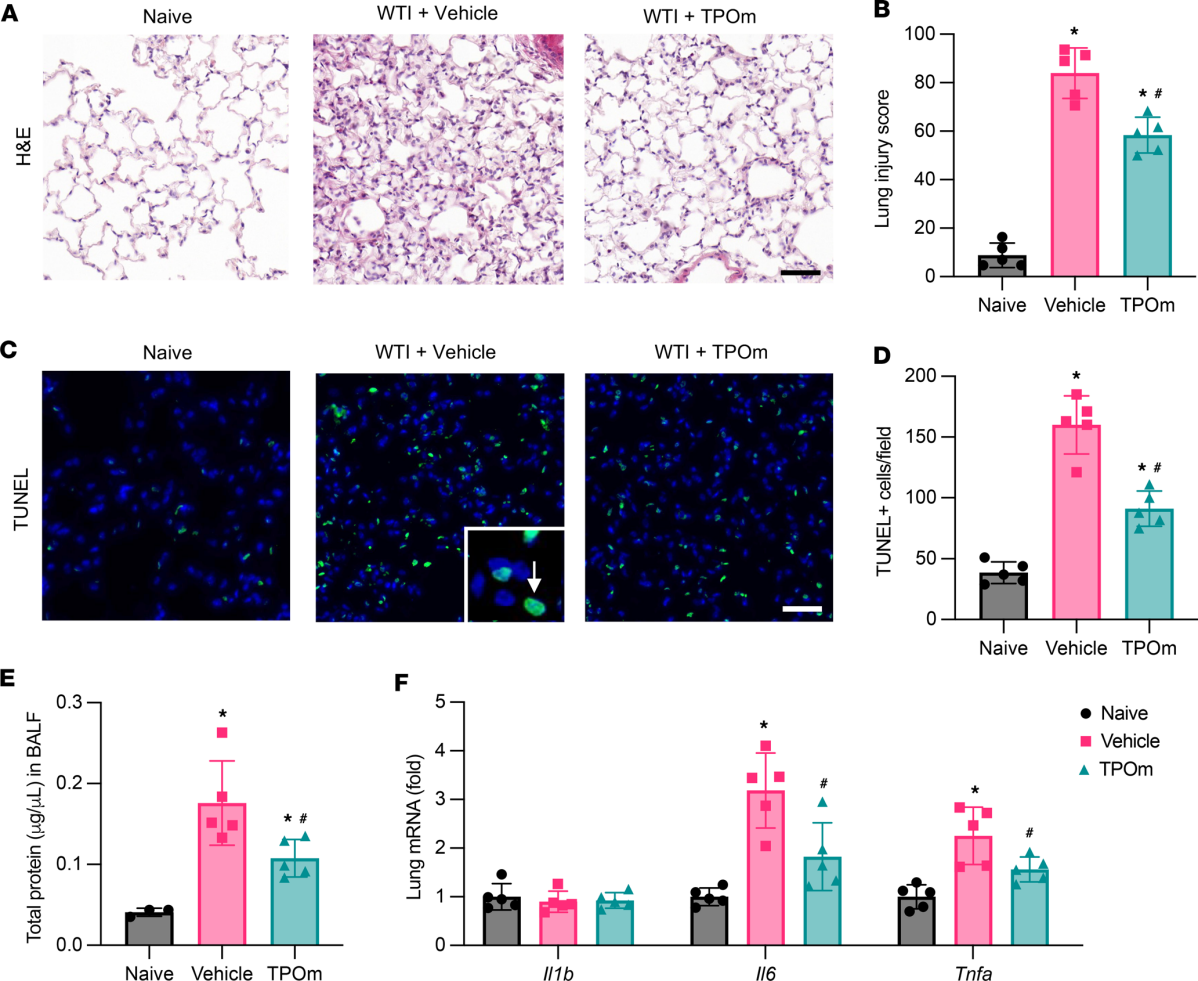
Effect of TPOm on radiation-induced lung damage, alveolar permeability, and inflammation in the mice 2 weeks after WTI. C57BL/6J mice were treated with PBS (Vehicle) or TPOm 24 hours before 16 Gy WTI. The lungs of naive and WTI mice were harvested 2 weeks after WTI. (**A**) Representative H&E staining of lung. Scale bar: 50 μm. (**B**) Histologic lung injury score judged by the H&E staining. (**C**) Representative TUNEL staining (green) in lung. Counterstained with DAPI (blue). Scale bar: 50 μm. The white arrow indicates TUNEL^+^ nucleus. (**D**) Quantification of TUNEL^+^ cells per field averaged over 5 microscopic fields/animal in each group. (**E**) Quantification of protein concentration in BALF. (**F**) Cytokine mRNA expression of *Il1b*, *Il6*, and *Tnfa* in lung, as determined by qPCR. The results of qPCR analysis are normalized with *Hprt1* as an internal control and are expressed as fold change compared with the naive group. Data are shown as mean ± SEM (*n* = 5/group). *P < 0.05 vs. naive and ^#^*P* < 0.05 vs. vehicle. Data were analyzed using nonparametric methods, using a 1-way ANOVA with Tukey’s test as post hoc comparison.

At 2 weeks after WTI in C57BL/6J mice, histological analysis of lung damage by H&E staining revealed that the vehicle-treated group exhibited extensive thickening of the alveolar septa and evidence of edema, while these were observed less in the TPOm-treated group (Figure 1A). Furthermore, the vehicle-treated group had more intra-alveolar cells than the naive and TPOm-treated groups (Figure 1A). Lung injury scoring from H&E staining reached 84 of 100 in the vehicle-treated group, while the score significantly reduced to 58 in the TPOm-treated group by 31% (Figure 1B). We also examined the number of apoptotic cells by TUNEL staining in lung tissues after WTI. The number of TUNEL^+^ cells in the vehicle-treated group was increased by 4.14-fold compared with the naive group, while it was significantly reduced by 43% with TPOm treatment (Figure 1, C and D). During radiation pneumonitis, the delicate blood-air barrier can be disrupted and cause an influx of fluid and proteins into the alveolar space (29). Measurement of the total protein exuded from vascular leakage in bronchoalveolar lavage fluid (BALF) at 2 weeks after WTI showed that the vehicle-treated group had protein content 4.25-fold higher than that of the naive group, while TPOm reduced the protein content by 43% compared with that in the vehicle-treated group (Figure 1E). Radiation increased mRNA expression of proinflammatory cytokines *Il6* and *Tnfa* in both the vehicle- and TPOm-treated groups; however, the expression of both cytokines was significantly reduced in the TPOm-treated group by 43% and 31%, respectively, compared with that in the vehicle-treated group (Figure 1F).

### TPOm inhibits neutrophil infiltration in the lungs of mice after WTI.

**Figure 2:**
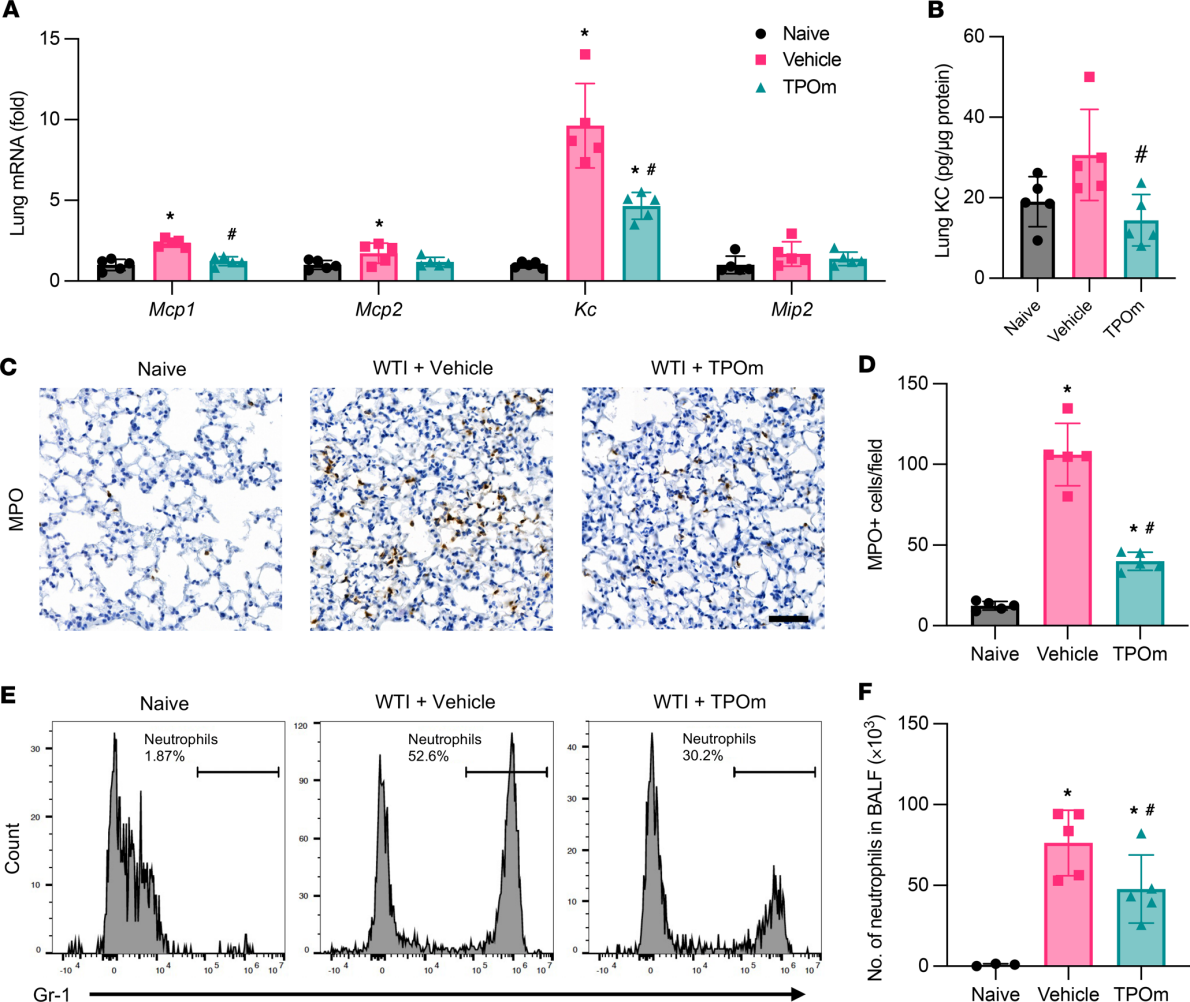
Effect of TPOm on radiation-induced neutrophil infiltration in the lungs of mice 2 weeks after WTI. C57BL/6J mice were treated with PBS (Vehicle) or TPOm 24 hours before 16 Gy WTI. The lungs of naive and WTI mice were harvested 2 weeks after WTI. (**A**) Chemokine mRNA expression of *Mcp1*, *Mcp2*, *Kc*, and *Mip2* in lung, as determined by qPCR. The results of qPCR analysis are normalized with *Hprt1* as an internal control and are expressed as fold change compared with the naive group. (**B**) Protein levels of KC in lung tissue lysate measured by ELISA. (**C**) Representative immunohistochemistry staining of MPO (brown) in lung. Counterstained with hematoxylin. Scale bar: 50 μm. (**D**) Quantification of MPO^+^ cell infiltrate in lung per field, averaged over 5 microscopic fields/animal in each group. (**E**) Representative flow cytometry contour plots of live/CD45^+^/Gr-1^+^ cells in BALF. (**F**) Quantification of total neutrophil number in BALF. Data are shown as mean ± SEM (*n* = 5/group). *P < 0.05 vs. naive and ^#^*P* < 0.05 vs. vehicle. Data were analyzed using nonparametric methods, using a 1-way ANOVA with Tukey’s test as post hoc comparison.

Heavy recruitment of immune cells to the damaged tissues contributes to local inflammation (29). We first examined the expression of specific chemokines that attract neutrophils, like KC, and macrophages, like MCP-1, in the lung 2 weeks after WTI. The analysis of chemokine mRNA expression revealed that *Mcp1* and *Kc* were upregulated in the vehicle-treated group by 2.35-fold and 9.36-fold, respectively, compared with the naive group (Figure 2A). The levels of these inflammatory markers were significantly reduced in the TPOm-treated group by 39% and 64% compared with those in the vehicle-treated group (Figure 2A). In parallel, the protein level of KC in lung was also significantly reduced in the TPOm-treated group to a level comparable to the naive group (Figure 2B). As KC is a major recruitment chemokine for neutrophils, myeloperoxidase (MPO) staining was used to assess neutrophil infiltration in the lung (Figure 2C). The number of neutrophils was significantly increased in the vehicle-treated group compared with the naive group, while it was significantly reduced in the TPOm-treated group by 59% (Figure 2D). Flow cytometric analysis also determined the number of neutrophils in the BALF (Figure 2E). Like the lung tissue, TPOm significantly decreased the number of neutrophils in the BALF compared with that in the vehicle-treated group by 37% (Figure 2F).

### TPOm attenuates activation of lung ECs after IR in vitro.

**Figure 3:**
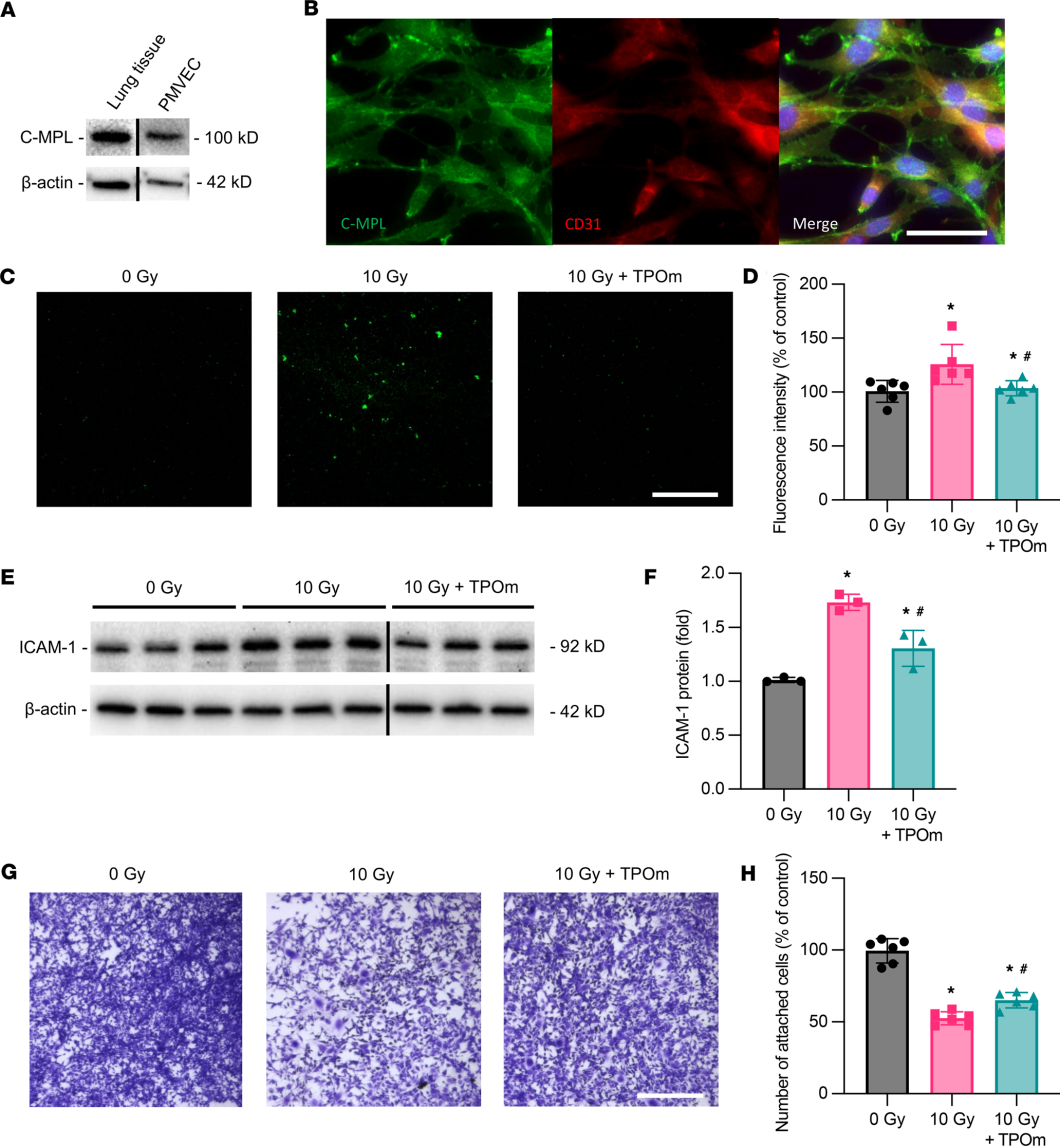
Effect of TPOm on neutrophil adherence and activation of irradiated mouse PMVECs. Mouse primary PMVECs were isolated from C57B/6J mouse and cultured. (**A**) Western blot analysis of c-MPL expression in lung tissue and PMVECs. β-Actin was used as loading control. (**B**) Immunofluorescence staining of c-MPL (green) and CD31 (red) in PMVECs. Counterstained with DAPI (blue). Scale bar: 40 μm. (**C**) Representative images of fluorescently labeled mouse neutrophils (green) coincubated on non- and irradiated PMVECs for 30 minutes and then washed with PBS. PMVECs were pretreated with PBS or TPOm 2 hours before irradiating at 10 Gy and then cultured for another 48 hours before adhesion assay. Adherent neutrophils were imaged by fluorescent microscopy. Scale bar: 100 μm. (**D**) Averaged fluorescence intensity of each group measured at an excitation and emission of 488/515 nm. The intensity of 0 Gy control is designated as 100% for comparison (*n* = 6/group). (**E**) Western blot images of ICAM-1 expression in mouse PMVECs from 0 Gy control and irradiated PMVECs at 10 Gy 48 hours after IR. The irradiated PMVECs were pretreated with PBS and TPOm 2 hours before IR. β-Actin was used as loading control. (**F**) Quantification of relative ICAM-1 protein levels compared with 0 Gy control set as 1 (*n* = 3/group). (**G**) Representative images of crystal violet staining of PMVECs from 0 Gy control and irradiated PMVECs at 10 Gy 24 hours after IR. The irradiated PMVECs were pretreated with PBS and TPOm 2 hours before IR. Scale bar: 100 μm. (**H**) Quantification of crystal violet staining after elution with methanol, followed by reading at 570 nm on a microplate reader. The OD reading of the 0 Gy control is designated as 100% for comparison (*n* = 6/group). Data are shown as mean ± SD. *P < 0.05 vs. 0 Gy and ^#^*P* < 0.05 vs. 10 Gy. Data were analyzed using nonparametric methods, using a 1-way ANOVA with Tukey’s test as post hoc comparison.

ECs play a crucial role in initiating the interaction with neutrophils for their entrance into tissue (30). We examined whether TPOm could directly regulate the behavior of lung ECs. c-MPL is the receptor of TPOm that mediates its biological activity in the cell. Western blot analysis revealed that c-MPL was well detected in lung tissue (Figure 3A). We further assessed the expression of c-MPL in the isolated mouse PMVECs and showed its expression (Figure 3A). We also performed immunofluorescence staining for c-MPL and CD31, an EC marker, in cultured mouse PMVECs and identified membrane localization of both receptors (Figure 3B). To evaluate the extent of EC activation, we performed a neutrophil adhesion assay. Coincubation of fluorescently labeled neutrophils for 30 minutes with mouse PMVECs irradiated at 10 Gy showed a 1.25-fold increase of adhered neutrophils compared with the nonirradiated mouse PMVECs, while pretreatment with TPOm decreased the adhered neutrophils to levels similar to nonirradiated mouse PMVECs (Figure 3, C and D). ICAM-1 in ECs is an important factor for neutrophil adhesion (30). The expression of ICAM-1 in PMVECs was increased 1.73-fold after IR, while the increased expression was inhibited in PMVECs with TPOm pretreatment (Figure 3, E and F). We also examined the effect of TPOm on the survival of mouse PMVECs after IR. By crystal violet staining, the number of PMVECs decreased 53% 24 hours after 10 Gy IR, while TPOm significantly increased the number of PMVECs by 13% compared with that in the vehicle-treated group (Figure 3, G and H).

### TPOm reduces lung fibrosis in mice after WTI.

**Figure 4:**
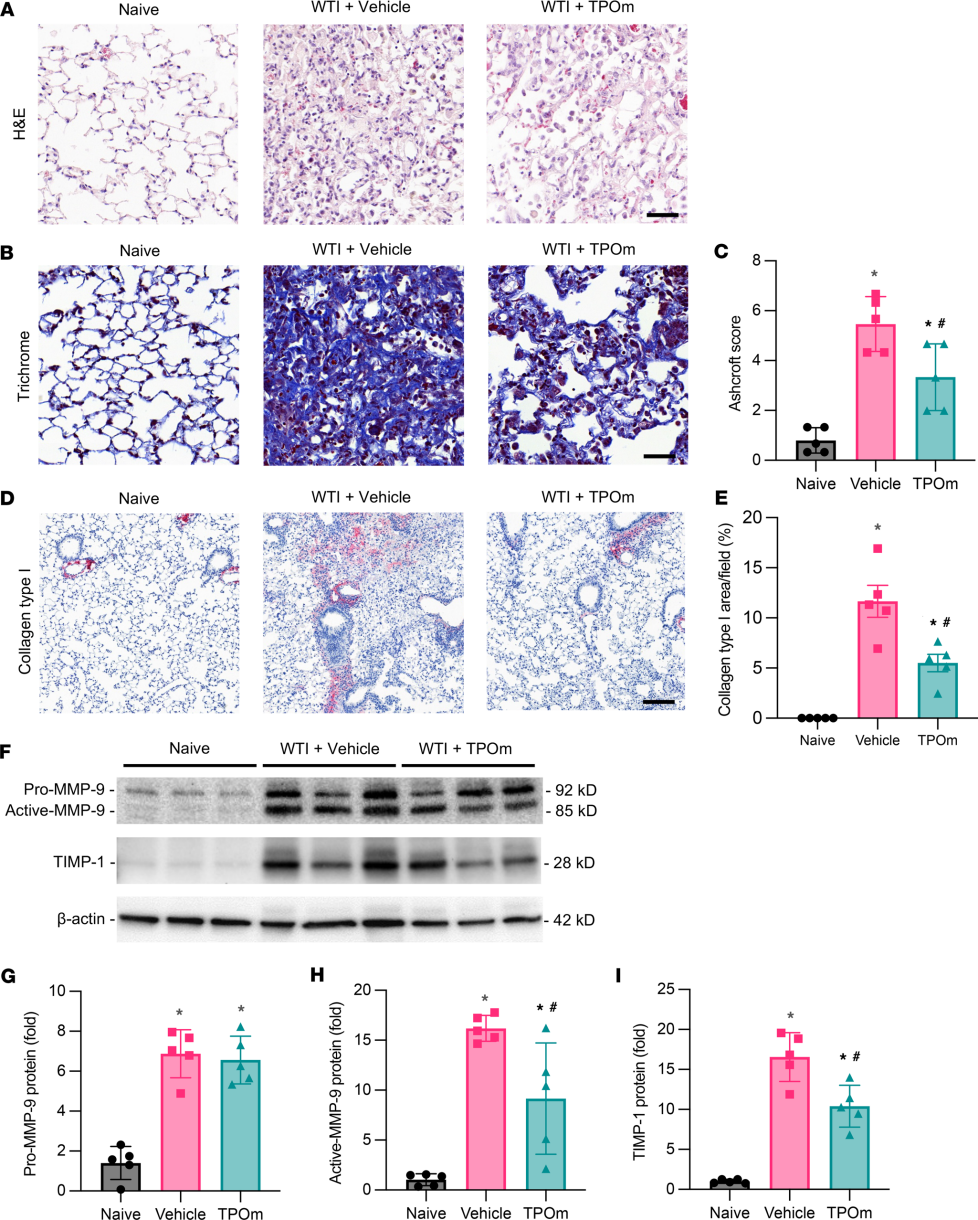
Effect of TPOm on radiation-induced fibrosis in the lungs of mice 7 months after WTI. C57BL/6J mice were treated with PBS (Vehicle) or TPOm 24 hours before 16 Gy WTI. The lungs of naive and WTI mice were harvested 7 months after WTI. (**A**) Representative H&E staining of lung. Scale bar: 50 μm. (**B**) Representative Masson’s trichrome blue staining of lung. Scale bar: 50 μm. (**C**) Aschroft score of Masson’s trichrome–stained sections. (**D**) Representative immunohistochemistry staining of collagen type I (red) of lung. Counterstained with hematoxylin. Scale bar: 125 μm. (**E**) Quantification of alveolar collagen type I immunohistochemistry. Reported as percentage of positive stained area per field averaged over 5 microscopic fields/animal in each group. (**F**) Representative Western blot images of precursor of MMP-9 (pro–MMP-9), active MMP-9, and TIMP-1 expression in lung. β-Actin was used as loading control. (**G**–**I**) Quantification of relative (**G**) pro–MMP-9, (**H**) active MMP-9, and (**I**) TIMP-1 protein levels compared with naive control set as 1. Data are shown as mean ± SEM (*n* = 5/group). *P < 0.05 vs. naive and ^#^*P* < 0.05 vs. vehicle. Data were analyzed using nonparametric methods, using a 1-way ANOVA with Tukey’s test as post hoc comparison.

A major characteristic of fibrosis in the lung is the replacement of functional alveoli with fibrotic, collagen-rich tissue (4). To examine the lung fibrosis, we harvested lung tissues 7 months after WTI for the analysis. The H&E staining of lung tissues in the vehicle-treated group exhibited immune cell infiltration, alveolar collapse, and thickening of the alveolar septa (Figure 4A). In contrast, the lungs of TPOm-treated mice showed less immune cell infiltration and alveolar collapse compared with the vehicle-treated group (Figure 4A). In Masson’s trichrome staining, the vehicle-treated group showed a large area stained with dense blue stain and obliterated alveoli, indicating robust collagen deposition, while there was less deposition in the TPOm-treated group (Figure 4B). Ashcroft scoring for semiquantitative analysis of Masson’s trichrome staining showed that the average of fibrosis score in the TPOm-treated group was significantly lower than that in the vehicle-treated group by 39% (Figure 4C). To be more specific about the type of collagens being aberrantly deposited in tissue remodeling, we examined the expression of collagen type I, which is located at blood vessels in the lung to support the structure (31). As expected, collagen type I was only detected in the area around the lung blood vessels in the naive group, while its location was extended to the alveolar area with a diffuse staining pattern in the vehicle-treated group (Figure 4D). In contrast, the expression of collagen type I in the TPOm-treated group was more limited in the vessel area compared with the vehicle-treated group (Figure 4D). The overall area stained with collagen type I in the lungs of the TPOm-treated group decreased by 53% compared with that in the vehicle-treated group (Figure 4E). We further examined the expression of MMP-9, which has been reported to correlate with the severity and prognosis of pulmonary fibrosis (32, 33). Western blot analysis revealed 2 bands denoting precursor of MMP-9 and active MMP-9 (Figure 4F). There were drastic increases in the expression of both forms of MMP-9 in the vehicle- and TPOm-treated groups after WTI compared with the naive group (Figure 4, F–H). However, the expression level of active MMP-9 in the TPOm-treated group decreased by 43% compared with that in the vehicle-treated group (Figure 4H). Extracellular matrix protein TIMP-1 is another factor in regulating the progression of lung fibrosis (34). Similar to MMP-9, the expression level of TIMP-1 was elevated in both the vehicle- and TPOm-treated groups compared with the naive group; however, its level decreased by 37% in the TPOm-treated group compared with that in the vehicle-treated group (Figure 4, F and I).

### TPOm decreases senescence in the lungs of mice after WTI.

**Figure 5:**
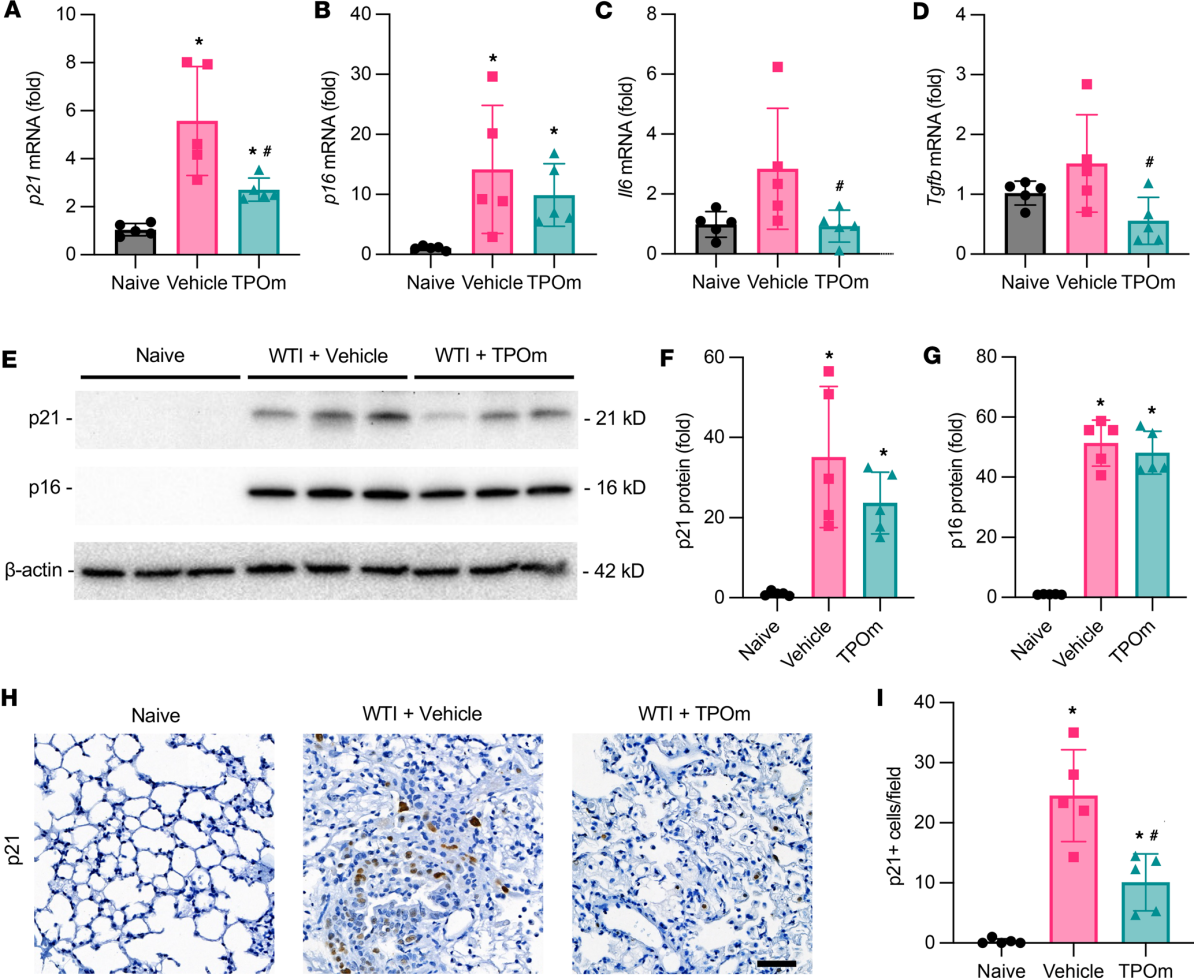
Effect of TPOm on radiation-induced senescence in the lungs of the mice 7 months after WTI. C57BL/6J mice were treated with PBS (Vehicle) or TPOm 24 hours before 16 Gy WTI. The lungs of naive and WTI mice were harvested 7 months after WTI. (**A**–**D**) The total RNA from lung tissue was isolated to analyze the mRNA levels of (**A**) *p21*, (**B**) *p16*, (**C**) *Il6,* and (**D**) *Tgfb* as determined by RT-qPCR. The results of qPCR analysis are normalized with *Hprt1* as an internal control and are expressed as fold change compared with the naive group. (**E**) Representative Western blot image of p21 and p16 of lung. β-Actin was used as loading control. (**F** and **G**) Quantification of relative (**F**) p21 and (**G**) p16 protein levels compared with naive control set as 1. (**H**) Representative immunohistochemistry staining of p21 (brown) in lung. Counterstained with hematoxylin. Scale bar: 50 μm. (**I**) Quantification of p21^+^ cells in lung per field averaged over 5 microscopic fields/animal in each group. Data are shown as mean ± SEM (n = 5/group). *P < 0.05 vs. Naive and ^#^*P* < 0.05 vs. Vehicle. Data were analyzed using nonparametric methods, using a 1-way ANOVA with Tukey’s test as post hoc comparison.

Senescent cell populations are major contributors to a profibrotic microenvironment in late RILI (14). Senescent cells characteristically express high levels of p21 and p16, resulting in cell cycle arrest (15). Senescent cells also have a high paracrine factor output; therefore, we examined the mRNA expression of 2 factors, *Il6* and *Tgfb*, identified in the SASP repertoire (14, 35). The mRNA levels of *p21*, *p16*, *Il6*, and *Tgfb* in lung tissue 7 months after WTI were elevated compared with the naive group (Figure 5, A–D). In contrast, TPOm treatment decreased the mRNA expression levels of *p21*, *p16*, *Il6*, and *Tgfb* by 51%, 39%, 77%, and 63%, respectively, compared with the vehicle-treated group (Figure 5, A–D). The protein expression levels of p21 also showed a reduction in the TPOm-treated group compared with the vehicle-treated group but didn’t reach a statistical significance (Figure 5, E and F). There was no difference in protein expression levels of p16 between the vehicle- and TPOm-treated groups (Figure 5, E and G). We also performed immunostaining against p21 in the lung tissues. The p21^+^ cells were barely detected in the naive group, while these cells were well observed in the fibrotic area of lung tissues in the vehicle-treated group (Figure 5H). In contrast, the number of p21^+^ cells per field in the TPOm-treated group significantly decreased by 59% compared with the vehicle (Figure 5, H and I).

### TPOm improves lung vascular structure in mice at the late phase after WTI.

**Figure 6:**
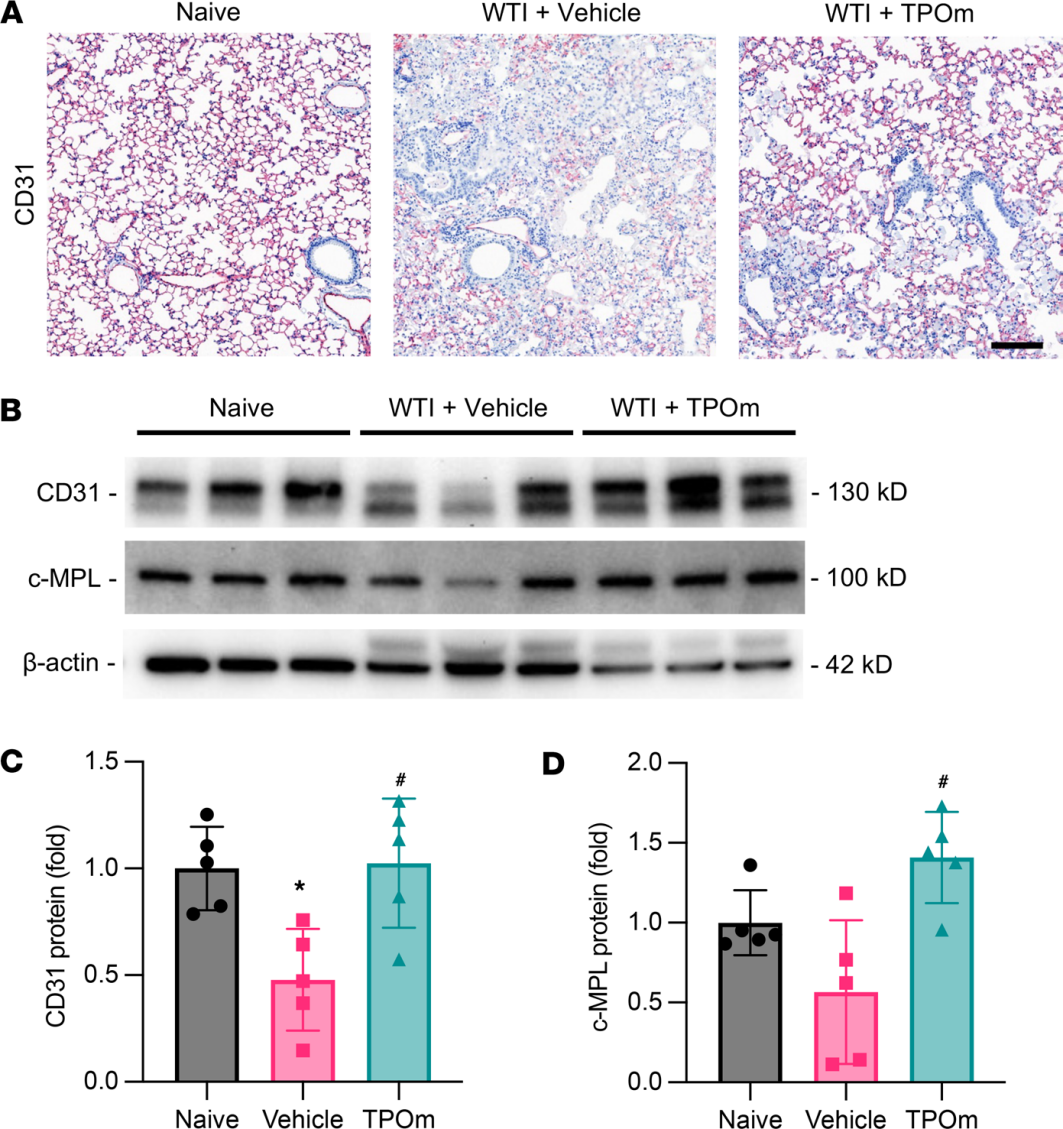
Effect of TPOm on radiation-induced changes in vascularity of the lungs of mice 7 months after WTI. C57BL/6J mice were treated with PBS (Vehicle) or TPOm 24 hours before 16 Gy WTI. The lungs of naive and WTI mice were harvested 7 months after WTI. (**A**) Representative immunohistochemistry staining of CD31 (magenta) in lung. Counterstained with hematoxylin. Scale bar: 125 μm. (**B**) Representative Western blot images of CD31 and c-MPL. β-Actin was used as loading control. (**C** and **D**) Quantification of relative (**C**) CD31 and (**D**) c-MPL protein levels compared with naive control set as 1. Data are shown as mean ± SEM (*n* = 5 per group). *P < 0.05 vs. naive and ^#^*P* < 0.05 vs. vehicle. Data were analyzed using nonparametric methods, using a 1-way ANOVA with Tukey’s test as post hoc comparison.

In a fibrotic lung, vascular collapse and loss of endothelial characteristics occur when damaged tissue is replaced by heavy deposition of extracellular matrix proteins (36). In the naive group, lung vascular ECs, identified by immunostaining against CD31, were homogenously distributed throughout the alveolar area (Figure 6A). The vascular loss was evident in the alveolar space of the vehicle-treated group 7 months after WTI, while the TPOm-treated group retained more CD31^+^ cells in the alveolar space than the vehicle-treated group (Figure 6A). By Western blot analysis of lung tissues at the same time point, CD31 expression was reduced by 55% in the vehicle-treated group compared with the naive group (Figure 6, B and C). The lung expression of CD31 in the TPOm-treated group was 2.12-fold higher than the vehicle-treated group, and there was no statistical difference to the naive group (Figure 6, B and C). Similar to the changes in CD31, the expression level of c-MPL in the vehicle was also reduced by 43% compared with the naive group (Figure 6, B and D). In contrast, the TPOm-treated group had significantly higher c-MPL expression by 2.51-fold than the vehicle-treated group (Figure 6, B and D).

### TPOm attenuates the loss of cardiopulmonary function in mice at the late phase after WTI.

**Figure 7:**
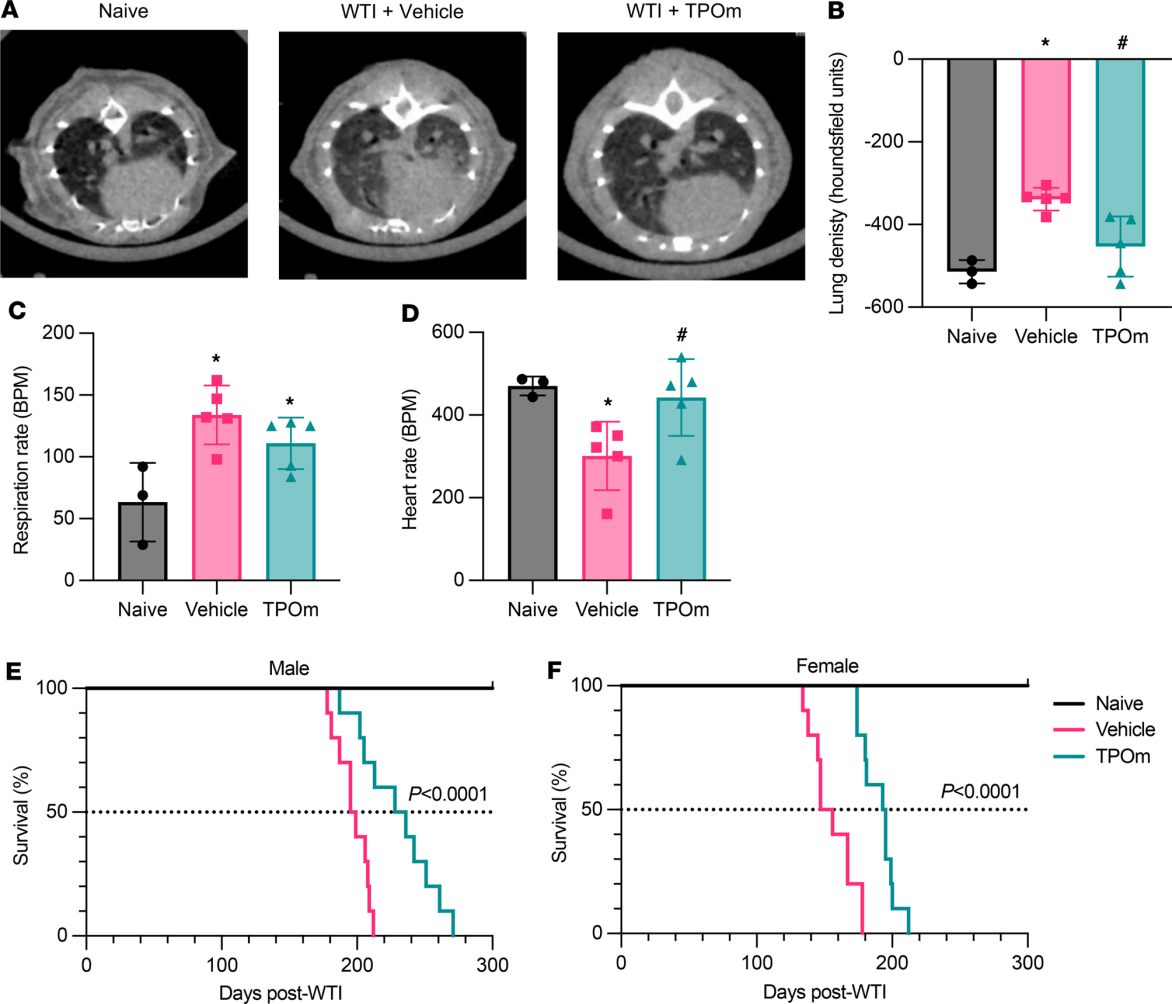
Effect of TPOm on lung-cardiac function of the mice 7 months after WTI and their survival after WTI. (**A**–**D**) C57BL/6J mice were treated with PBS (Vehicle) or TPOm 24 hours before 16 Gy WTI. The naive and WTI mice were scanned 7 months after WTI. (**A**) Representative μCT thoracic cross sections. (**B**) Quantification of radiodensity of 3D contoured μCT lung images. (**C**) Respiration rate of each mouse reported as breaths per minute (BPM). (**D**) Heart rate of each mouse reported as beats per minute (BPM). Data are shown as mean ± SEM (*n* = 3–5/group). *P < 0.05 vs. naive and ^#^*P* < 0.05 vs. vehicle. (**E** and **F**) C57BL/6J mice were treated with PBS or TPOm 24 hours before 18 Gy WTI. The survival time of (**E**) male and (**F**) female mice after WTI with Kaplan-Meier plot. The reported *P* value is from log rank comparing survival curves between vehicle and TPOm. μCT, breath rate, and heart rate data were analyzed using nonparametric methods, using a 1-way ANOVA with Tukey’s test as post hoc comparison. Survival data were analyzed using Kaplan-Meier analysis.

Lung density, respiratory rate, and heart rate are often measured to monitor the decline of pulmonary health in patients in the late stage of RILI. We performed micro-computed tomography (μCT) imaging to measure the lung density of mice 7 months after WTI (Figure 7A). Compared with naive mice as the baseline, the radiodensity signal in the lungs of the vehicle-treated group was 1.34-fold higher (Figure 7B). In contrast, the TPOm-treated group had significantly lower radiodensity, by 25%, compared with the vehicle-treated group (Figure 7B). At the time of μCT scanning, the respiratory rate of each mouse was measured. The breath rate of the vehicle-treated group was 2.13-fold higher than that of the naive group, while TPOm treatment slightly decreased the breath rate compared with the vehicle-treated group (Figure 7C). We also evaluated cardiac function by electrocardiography. Compared with the naive group, the vehicle-treated group presented with a much lower heart rate (Figure 7D). However, the TPOm-treated group had a significantly higher heart rate than the vehicle-treated group (Figure 7D).

### TPOm prolongs the survival time of mice after WTI.

Survival is an important endpoint in evaluating efficacy of drugs for radioprotection (37). The median survival time in the vehicle-treated male mice was 197 days (95% CI, 0.3232–2.231) while the median survival time in the TPOm-treated male mice was 232 days (95% CI, 0.4483–3.094), which was significantly longer than the vehicle-treated male mice (Figure 7E). The median survival time in the vehicle-treated female mice was 151 days (95% CI = 0.3250 to 1.876) while the median survival time in the TPOm-treated female mice was 194 days, which was significantly longer than the vehicle-treated female mice (95% CI, 0.5330–3.077) (Figure 7F).

### TPOm redistributes subpopulations of lung capillary ECs in mice at the pneumonitis phase after WTI.

**Figure 8:**
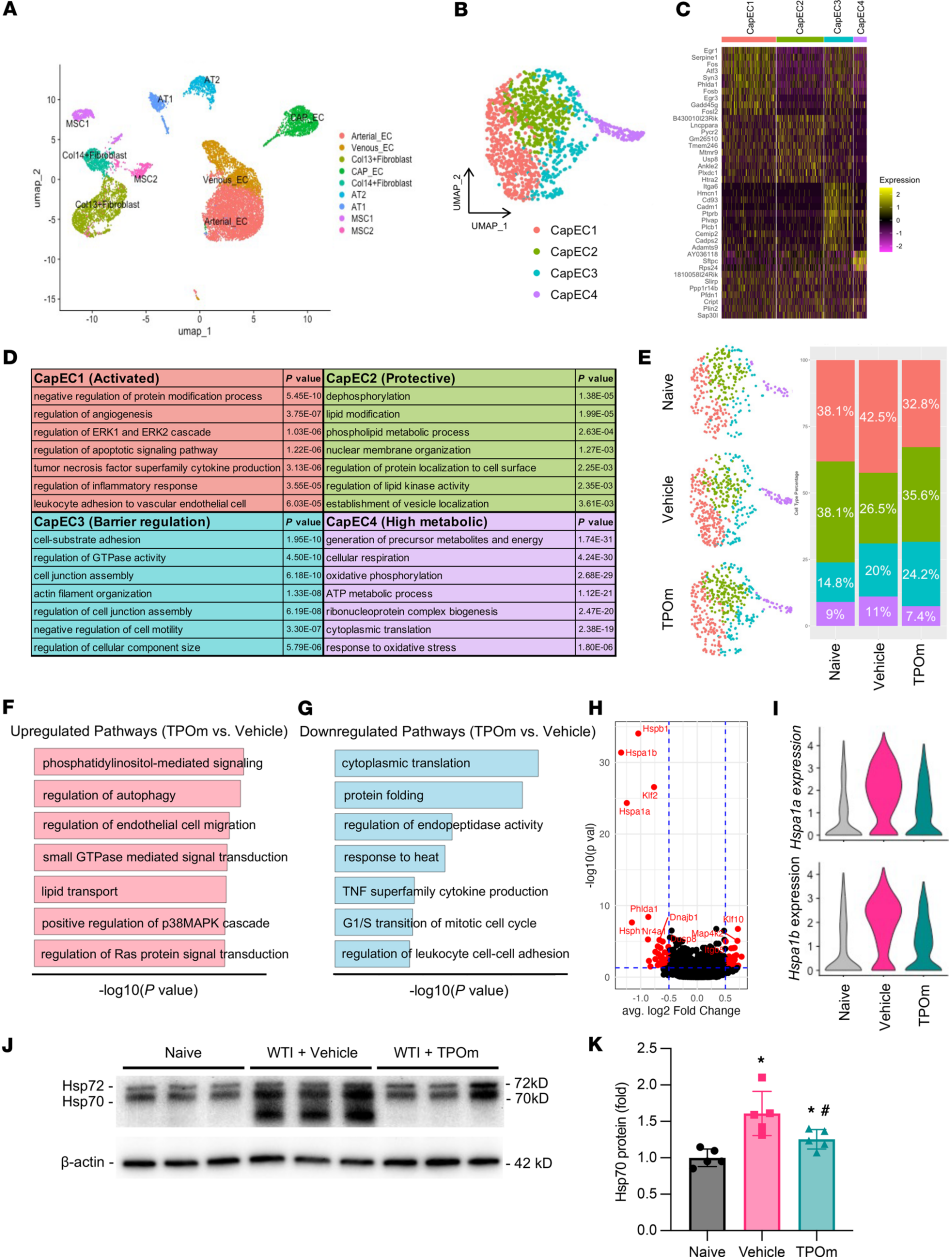
Effect of TPOm on radiation-induced phenotypic changes of capillary endothelial cells in lungs of mice 2 weeks after WTI. C57BL/6J mice were treated with PBS (Vehicle) or TPOm 24 hours before 16 Gy WTI. Lungs of naive and WTI mice were harvested 2 weeks after WTI, and nonhematopoietic lung cells (CD45^-^) were isolated for scRNA-Seq analysis. (**A**) 2D UMAP projection of 12,238 individual lung cells isolated across treatment groups. Arterial_EC, arterial EC; Venous_EC, venous EC; Col13+Fibroblast collagen type 13^+^ fibroblasts; CAP_EC, capillary EC; Col14+Fibroblast, collagen type 14^+^ fibroblasts; AT2, alveolar type 2 cells; AT1, alveolar type 1 cells; MSC1, mesenchymal stromal cells 1; MSC2, mesenchymal stromal cells 2. (**B**) 2D UMAP projection of 1,287 lung capillary ECs (CapECs). Different colors denote different clusters: CapEC1, CapEC2, CapEC3, and CapEC4. (**C**) Heatmap of the most differentially expressed genes in each CapEC cluster. Color bars represent gene expression in log_2_ scale. (**D**) Enriched biological processes based on Gene Ontology analysis and enriched genes found in each cluster. *P* values are indicated to the right of each enriched term found in that particular cluster. (**E**) 2D UMAP projection of CapECs split among naive, vehicle-, and TPOm-treated lung cells. Each cluster’s distribution by percentage is iterated by treatment condition. (**F** and **G**) Biological process (**F**) upregulated and (**G**) downregulated in TPOm vs. vehicle. Length of bar coordinates with –log_10_ *P* value. (**H**) Volcano plot of differentially expressed genes of TPOm-treated vs. vehicle-treated cells. (**I**) Violin plots of *Hspa1a* and *Hspa1b* across each treatment condition. (**J**) Representative Western blot images of Hsp70 and Hsp72 in lung. β-Actin was used as loading control. (**K**) Quantification of relative Hsp70 protein levels compared with naive control set as 1. Data are shown as mean ± SEM (*n* = 5/group). *P < 0.05 vs. naive and ^#^*P* < 0.05 vs. vehicle. Data were analyzed using nonparametric methods, using a 1-way ANOVA with Tukey’s test as post hoc comparison. Statistical test of differential expression in scRNA-Seq analysis was completed using MAST.

As demonstrated earlier, TPOm attenuated the activation of mouse PMVECs induced by IR and decreased inflammation in mice after WTI, inhibiting subsequent fibrosis. To further understand the effect of TPOm on the transcriptional landscape of the lung cells at the radiation pneumonitis phase, we performed scRNA-Seq analysis of lung tissues from mice 2 weeks after WTI. The isolated lung cells were subjected to flow cytometry to exclude the hematopoietic (CD45^+^) and dead cells before sequencing. The UMAP projection using the Seurat package revealed 9 transcriptionally distinct clusters or cell populations in the lung after data integration and filtering from all 3 groups, including the naive, vehicle, and TPOm treatment groups (Figure 8A). The differential gene expression profile and list of genes for generating each cluster are shown in the supplemental materials (Supplemental Figure 1A and Supplemental Table 1; supplemental material available online with this article; https://doi.org/10.1172/jci.insight.181330DS1). Around 5,000 lung cells in each mouse group were sequenced, and the proportion of each cluster in these 3 groups was well maintained (Supplemental Figure 1, B and C). Although the number of different cell populations in the lung had been recovered from radiation injury 2 weeks after WTI, there might be phenotypical differences in each cluster among the groups.

We first performed the clustering analysis in the capillary EC (CapEC) population, which is the source of mouse PMVECs used in the in vitro study. Four subpopulations, CapEC1–4, were identified within the CapEC cluster according to their differences in the gene expression profile (Figure 8, B and C, and Supplemental Table 2). By analyzing their enriched pathways, we assigned the phenotypic function of each CapEC subpopulation as follows: CapEC1 as an activated population, enriched for processes of protein modification, ERK cascade, apoptotic signaling, cytokine production, and leukocyte interaction pathways; CapEC2 as a protective population, enriched for processes of dephosphorylation, membrane organization, protein localization, and lipid kinase activity pathways; CapEC3 as a barrier regulation population, enriched for processes of matrix adhesion, cell junction assembly, and GTPase activity pathways; and CapE4 as a high metabolic population, enriched for processes of generation of metabolites and energy, cellular respiration, oxidative phosphorylation, and oxidative stress pathways (Figure 8D). Lists of all the identified pathways in each CapEC subpopulation are provided in Supplemental Tables 3–6. CapEC1 and CapEC2 populations were more abundant than CapEC3 and CapEC4 populations (Figure 8E). The percentage of energetic CapECs, including CapEC1 and CapEC4, increased in the vehicle-treated group compared with the naive group, while the TPOm-treated group decreased their percentage compared with the vehicle-treated group (Figure 8E). Correspondingly, TPOm increased the regulatory CapECs, including CapEC2 and CapEC3, compared with the vehicle-treated group (Figure 8E).

To understand by which mechanisms TPOm regulated the phenotypic shift of CapECs in the irradiated lung, we compared the differences in pathways and gene expression in the CapEC cluster between vehicle- and TPOm-treated groups. The pathways involved in phosphatidylinositol-mediated (PI-mediated) signaling, small GTPase transduction, and positive regulation of p38MAPK cascade assembly were upregulated in the TPOm-treated group compared with the vehicle-treated group (Figure 8F and Supplemental Table 7), while the pathways involved in protein folding, TNF superfamily production, and leukocyte adhesion were downregulated in the TPOm-treated group compared with the vehicle-treated group (Figure 8G and Supplemental Table 8). The difference in the gene expression between vehicle- and TPOm-treated groups was shown in a volcano plot (Figure 8H). *Hspa1a* and *Hspa1b* encoding heat shock protein 70 (Hsp70) and *Klf2*, a transcription factor for endothelial nitric oxide synthase (eNOS), showed the highest difference between these two groups (Supplemental Table 9). The violin plot further demonstrated that a majority of CapECs in the vehicle-treated group had high expression of *Hspa1a* and *Hspa1b* compared with the naive and TPOm-treated groups (Figure 8I). The expression levels of Hsp70, as determined by Western blot analysis, in mouse lungs at 2 weeks after WTI also showed that the vehicle-treated group had 1.61-fold higher expression than the naive group (Figure 8, J and K). At the same time, TPOm treatment decreased Hsp70 expression by 26% compared with that in the vehicle-treated group (Figure 8, J and K). Of note, the degraded Hsp70 was detected in the vehicle-treated group, but not in the naive and TPOm-treated groups (Figure 8J). Moreover, the mRNA expression of *Hspa1a* and *Hspa1b*, as determined by qPCR, also showed an upregulation in the cultured mouse PMVECs 4 days after IR compared with the naive group and was inhibited by TPOm treatment (Supplemental Figure 2).

## Discussion

Normal tissue toxicity is a major concern following radiotherapy for patients with lung cancer. There continue to be challenges to targeting key cell types contributing to the pathogenesis of RILI and, thus, the development of new therapeutic agents for its treatment. Using the WTI mouse model in this study, we evaluated the targeting and potential of TPOm as a radioprotector to ameliorate RILI. We have demonstrated that TPOm pretreatment decreases lung injury score, apoptosis, pulmonary vascular leakage, and inflammatory signaling in the WTI mice at radiation pneumonitis phase. We have also demonstrated that TPOm inhibits the production of chemokine KC and recruitment of neutrophils to the lung and the BALF. We further identified c-MPL expression in the lung and isolated mouse PMVECs. Consistent with in vivo findings, in vitro cultures of irradiated mouse PMVECs treated with TPOm showed less propensity to adhere to isolated neutrophils and lower expression of ICAM-1. At the subsequent late fibrosis phase, WTI mice treated with TPOm showed a reduction of collagen deposition, senescence, and vascular loss in the lung. We have further demonstrated that TPOm improves the lung density, respiration rate, and heart rate of WTI mice, leading to an increase in survival time of WTI male and female mice. In the scRNA-Seq analysis of nonhematopoietic lung cells, TPOm shifted the phenotypes of CapEC subpopulations in the WTI mice at the radiation pneumonitis phase from energetic to regulatory populations. Taken together, treatment of TPOm prior to radiation provided short- and long-term lung protection from RILI pathologies by modulating EC activation.

Mouse models of thoracic IR using various mouse strains have been developed to recapitulate the clinical outcomes of RILI in humans (28). The C57BL/6J mouse strain is commonly used in investigations involving WTI for RILI, which presents in two distinct phases after exposure: pneumonitis and fibrosis. Other mouse strains, such as CBA and C3H, exhibit acute radiation pneumonitis of RILI but do not further develop fibrosis (28). Moreover, mortality in C57BL/6J mice during the pneumonitis phase is lower than that in those other strains (38). As C57BL/6J mice can be observed through late time points to capture RILI progression fully, this strain is a valuable tool in evaluating the effectiveness of therapeutic agents to treat RILI (38). Like RILI in humans, elevated inflammatory signaling in the mice after IR leads to aberrant infiltration of activated neutrophils and macrophages into lung tissue (2). Other studies have shown that chronic inflammation heavily contributes to the pathogenesis of pulmonary fibrosis in mice after IR (39).

There is strong evidence indicating that dysfunction and loss of ECs contribute to radiation-induced toxicities in multiple organs (40). Constitutively activated ECs after radiation exposure not only cause vascular injury, but also prime for chronic inflammation (10). One mechanism by which TPOm can protect the lung after IR is through direct interaction with ECs to limit inflammation and subsequent fibrosis, as demonstrated here. It has been reported that human umbilical vein ECs express c-MPL to mediate TPO activity for stimulating prosurvival and proangiogenic PI3K and AKT pathways (26). We have demonstrated that TPOm protects mouse PMVECs, which express c-MPL from radiation-induced cell death. We have also observed a reduction of apoptosis in the lung by TPOm. Whether TPO-mediated protection is specific to ECs needs further investigation. A study has demonstrated that WTI can induce apoptosis in both ECs and epithelial cells in the lung (41). PMVECs also respond to TPOm by limiting their interaction with neutrophils through inhibiting ICAM-1 expression in PMVECs. The literature supports that ICAM-1 is required for neutrophil traffic in lung during an inflammatory response (30). The inhibition of EC activation by TPOm demonstrated in our in vitro study is also reflected in our in vivo observations of lower neutrophil infiltration, vascular permeability, and inflammatory response in lungs of WTI mice treated with TPOm, leading to improved lung structure. Neutrophil influx into lung interstitium and air spaces is a defining characteristic of radiation-induced early inflammation (29). The integrity of the epithelial/endothelial barrier is fundamental to preventing alveolar edema, leukocyte infiltration, and maintenance of alveolar fluid (13). A rescue of alveolar permeability has been shown to coordinate with reduced inflammation and improved histopathological outcomes in another RILI study (42). As demonstrated in TPOm activity, targeting inflammation through inhibiting neutrophil activity, chemotaxis, and infiltration has been a successful strategy in resolving chronic inflammation and reduce fibrosis in mice after WTI (43, 44). We did not examine the lungs of naive mice 2 weeks after TPOm treatment; however, our previous studies showed that TPOm increases the platelet count of naive mice, peaking on days 6–10 after injection and returning to baseline on day 14 (20, 21). Furthermore, there was no severe adverse effect observed in the animal toxicity study of TPOm with single-dose injection (45).

By performing scRNA-Seq analysis, we further clustered CapECs in 4 different phenotypic subpopulations, named activated (CapEC1), protective (CapEC2), barrier regulation (CapEC3), and high metabolic (CapEC4). This study is the first to our knowledge to describe heterogenic populations of CapECs in the lung after radiation exposure. WTI increased the energetic CapEC populations (activated and high metabolic), while TPOm decreased the energetic CapEC populations toward regulatory CapEC populations (protective and barrier regulation). In the CapEC1 signature genes, early growth response protein 1 (*Egr1*), plasminogen activator inhibitor 1 (*Serpine1*), and a proto-oncogene, *Fos* have been implicated in endothelial injury and inflammatory response (46). The deficiency of *Egr1* in ECs decreased vascular inflammation in a model of atherosclerosis (47). In the CapEC2 signature genes, aryl hydrocarbon receptor (*Ahr*) is known as an environmental sensor and has a protective role in endothelium barrier, where upon injury there is loss of AHR signaling (48, 49). Whereas insulin receptor substrate-1 (*Irs1*) overexpression in ECs has been shown to support angioblast differentiation and wound healing (50). In the CapEC3 signature genes, expression of cluster of differentiation 93 (*Cd93*), bone morphogenetic protein 6 (*Bmp6*), and integrin subunit α 6 (*Itga6*) strengthens the endothelial barrier phenotype. CD93 helps maintain endothelial barrier function by stabilizing vascular endothelial–cadherin at cell junction sites (51). *Bmp6* has been implicated as a mediator of angiogenesis and lung vascular permeability associated with acute lung injury (52). Integrins play an important role in regulating vascular permeability; in particular, *Itgb1* and *Itgb4* have been shown to protect from vascular leakage in acute lung injury (53, 54). Although there is no direct evidence showing *Itga6* involvement in regulating the endothelial barrier, it can form heterodimers with *Itgb1* and *Itgb4* in ECs (55). As we demonstrated, the high percentage of the CapEC3 population in the TPOm-treated group reflects on the lower total protein leaked to BALF 2 weeks after WTI. The CapEC4 signature genes include two ribosomal proteins, ribosomal protein S24 (*Rsp24*) and mitochondrial ribosomal protein S33 (*Mrps33*). The primary function of ribosomes is for protein synthesis, which has been shown to regulate cell growth and metabolism (56). This scRNA-Seq analysis also validated the in vitro mouse PMVEC study showing that TPOm can modulate EC activity. These scRNA-seq data provide rich information to the broader context of how CapEC subpopulations respond and contribute to RILI.

We have also identified several signal transduction pathways in CapECs that are upregulated by TPOm treatment from the scRNA-Seq analysis, such as PI-, GTPase-, p38MAPK-, and Ras-mediated signaling. GTPase-mediated signaling has been shown to regulate the endothelial barrier and EC cytoskeleton to maintain homeostatic vascular permeability (12). Correspondingly, TPO has been shown to activate the Ras, MAPK, and PI3K singling pathways for megakaryocytic differentiation (57, 58). In the meantime, the pathways involved in regulating TNF superfamily cytokine production and leukocyte cell-cell interaction are downregulated by TPOm treatment, which is consistent with our results shown in lung tissue of mice 2 weeks after WTI and an in vitro mouse PMVEC study. By comparing the differential gene expression in the lung CapECs between vehicle- and TPOm-treated mice, we found that expression of Hsp70 was significantly downregulated in TPOm-treated group, which may play a role in the phenotypic shift of CapECs. The change of Hsp70 expression was further confirmed in the lungs of naive, vehicle-, and TPOm-treated mice 2 weeks after WTI as well as in irradiated mouse PMVECs treated with TPOm. Hsp70 proteins are molecular chaperones that assist protein folding processes, especially when cells are under stress conditions (59). As expected, the expression of Hsp70 in the lung is highly elevated after WTI, representing ongoing proteostatic stress. The lower Hsp70 expression in the TPOm-treated group is potentially due to more CapECs recovering from the IR stress than in the vehicle-treated group. Moreover, Hsp70 can be released extracellularly and serve as damage-associated molecular patterns with immunomodulatory functions by stimulating innate and adaptive immunity (60). Hsp70 also has autocrine functions on ECs to stimulate IL-6 and ICAM-1 expression (61). The extracellular Hsp70 can contribute to the inflammation that we observed in the lungs of mice 2 weeks after WTI. TPOm downregulated the heat shock repertoire, corresponding with previous studies demonstrating that Hsp90 inhibitors protect and restore the EC barrier integrity (62, 63). Furthermore, *Klf2*, a transcription factor for eNOS, was also found to be downregulated in the TPOm-treated group. eNOS upregulation has shown to be a part of LPS-induced lung EC injury (11). Another differentially expressed gene was integrin-β5 (*Itgb5*), which is upregulated in the TPOm-treated mice compared with the vehicle-treated group. *Itgb5* plays an important role for lung vascular homeostasis and endothelial survival (64). More investigations are being conducted to identify other molecules regulated by TPOm to control EC phenotypes and other lung cell populations. Although the CapEC cluster was the focus of this study, the effect of IR and TPOm treatment on other cell clusters will be analyzed in a follow-up study.

In the pathogenesis of RILI, the chronic release of inflammatory mediators from damaged ECs and immune cells encourages cells to enter a senescent state (14). Investigations using WTI have found that limiting inflammation in the early phase of RILI correlated with less appearance of senescence and fibrosis (65). In our study, irradiated mice showed increased expression of senescence markers p16 and p21. With TPOm treatment, *p21* expression was significantly lower than that of vehicle treatment. While mitotically inactive, these cells continuously release SASP factors to the alveolar microenvironment that encourage neighboring cells to become senescent and fibroblast proliferation (14). For the vehicle-treated group in this study, mRNA expression of proinflammatory *Il6* and profibrotic *Tgfb* were increased while TPOm treatment resulted in the reduction of their expression levels, similar to the naive group. TGF-β has been heavily implicated in developing fibrosis in multiple organs (16). A previous study reported that inhibiting senescence was associated with mitigating IR-induced lung fibrosis (66). The data here suggest that TPOm treatment attenuated radiation-induced senescence and limited a SASP repertoire-filled microenvironment, resulting in less fibrosis.

After radiation exposure, early injury changes the microenvironmental milieu and slowly progresses the replacement of functional endothelium with fibrotic, collagen-rich lesions (3). Consistent with previous literature, the lungs of WTI mice at 7 months present fibrotic lesions and alveolar thickening, as demonstrated by Masson’s trichrome blue staining in this study. The fibrotic areas also showed increased atypical expression of type 1 collagen in the distal alveolar regions of the lung, while with TPOm treatment its expression stayed primarily localized near airways. The histological observations are reflected in the elevation of MMP-9 and TIMP-1 expression in WTI mice, while their expression was attenuated by TPOm treatment. Alterations to the MMP/TIMP axis are important factors for the development of lung fibrosis after IR (18). It has been reported that downregulation of TIMP-1 and reduced lung fibrosis in mice can result from neutrophil depletion during the inflammatory phase of the bleomycin injury model (67). Consistent with the literature, here TPOm inhibited the neutrophil infiltration into the lungs of mice 2 weeks after WTI. With less inappropriate collagen deposition in the WTI mice treated with TPOm, there are more areas of functional tissue, as shown by the retention of CD31 expression and vascularity of the lungs. With radiation-induced pulmonary hypertension being a major comorbidity in RILI, it is important to have long-term retention of vasculature after damage (68). The assessment of lung function, including lung density and breath rate, reflects the degree of fibrosis in naive, vehicle-, and TPOm-treated mice. Due to the loss of surface area of the blood-air interface in the lung during fibrosis, vehicle-treated animals have more respirations to supply their tissues with the same amount of oxygen as naive or TPOm-treated animals. The better maintenance of lung function in the TPOm-treated mice also reflects on the prolonged survival time of WTI male and female mice. In addition, the heart rate in WTI mice was decreased in compared with the naive group, suggesting that heart failure may also contribute to the mortality of WTI mice. The reduction of heart rate after WTI has also been observed in a rat model, which is due to severe right ventricular enlargement with the impaired left ventricular function and cardiac output (69). TPOm treatment significantly increased the heart rate of the WTI mice. Whether TPOm has a direct effect on protecting ECs in the heart from IR needs further investigation.

This study demonstrates that TPOm can provide radioprotection against the downstream histopathological and functional deterioration in the lung following exposure to a high acute radiation dose. Unlike other preclinical mitigators targeting general outcomes of RILI, TPOm can act on early inflammation phase by modulating CapEC activation and stress response, subsequently reducing the senescence and fibrosis in the lung. In recent years, the landscape of therapeutic intervention for radioprotection and mitigation has remained confined to acute pathologies, while patients experiencing lung fibrosis and vascular injury continue to be clinically underserved. The results found in this study open a therapeutic avenue of study focused on endothelial protection after radiation injury. Moreover, preclinical and clinical studies indicated that TPOm is safe for human use (45, 70). It warrants further development of TPOm as a therapeutic agent for treating RILI.

## Methods

### Sex as a biological variable.

Our defined endpoint studies exclusively examined male mice to identify the TPOm mechanism. Our murine survival studies examined male and female animals, and similar findings are reported in both sexes.

### Animals.

The 9- to 12 week-old male and female C57BL/6J mice were obtained from The Jackson Laboratories.

### IR.

Mice were anesthetized with ketamine/xylazine and positioned prone in a lead shielded jig that exposed the thorax through a 2 cm opening. WTI was done using a CIX-3 orthovoltage X-ray cabinet (Xstrahl) at 300 kVp, 10 mA with 1 mm Cu filtration. Doses of 16 Gy (sublethal, *n* = 5/time point/group) or 18 Gy (lethal, *n* = 10/group) were administered at a dose rate of 1.95 Gy/min, between 0900 and 1100 hours. Cells were irradiated in an open field with same operating characteristics.

### TPOm administration.

TPOm (JNJ-26366821) from Johnson & Johnson was reconstituted in PBS at 200 μg/mL. Mice were randomly assigned to 3 groups: nonirradiated mice (naive), WTI mice treated with PBS (vehicle), and WTI mice treated with TPOm. A single subcutaneous injection of vehicle or TPOm was administered 24 hours prior to WTI. TPOm-treated mice were dosed at 1 mg/kg based on a previous study (20).

### Health status monitoring and survival.

The condition of WTI-treated mice was assessed, and mice were weighed weekly after IR. For the survival study, mice were monitored for 300 days. Some mice developed radiation dermatitis and were treated with Neo-To-Go (Johnson & Johnson) every 2–3 days.

### Histology and tissue collection.

Mice were euthanized by isoflurane inhalation for lung tissue collection at 2 weeks and 7 months after WTI. The right lung was inflated and fixed with 200 μL of 10% formalin, embedded in paraffin, and sectioned at 5 μm. Slides were stained with H&E, and images were acquired in alveolar spaces (equidistant from the center large vessels and pleura surface) and scored according to an established American Thoracic Society lung injury scoring system by a blinded investigator (71). Fibrosis was evaluated using the One-step Trichrome Stain Kit (StatLab) and qualitatively evaluated by a blinded investigator using a modified Ashcroft score (72). Deparaffinized sections were subjected to antigen retrieval, blocking, and primary antibody incubation for immunohistochemistry against p21 (Abcam, ab188224, clone EPR18021), MPO (Abcam, ab208670, clone EPR20257), CD31 (Cell Signaling, 77699T, clone D8V9E), and collagen type I (Cell Signaling, 72026, clone E8F4L). Immunoreactivity was visualized by ImmPACT histochemistry (Vector Labs) with hematoxylin counterstaining. Slides were imaged using a P250 High-Capacity Slide Scanner (3DHistech). Quantification of MPO^+^ and p21^+^ cells and collagen type I^+^ area in lung tissue was performed with ImageJ software (NIH). Data were quantified from 3 fields of per animal and 5 animals per group.

### Bronchoalveolar lavage.

At harvest, BALF was collected from mouse lungs 2 weeks after WTI as described previously (73). A 22-gauge venous catheter was inserted into the trachea, and PBS was instilled into the lung at 0.5 mL for 60 seconds followed by aspiration. Bradford assay was performed on BALF using Protein Dye Reagent (Bio-Rad).

### Flow cytometry of BALF cells.

Following BALF collection, cells were pelleted, resuspended in FACS buffer (PBS containing 2% FBS and 1 mM EDTA), and counted. BALF cells were stained with Zombie NIR (BioLegend), followed by Fc block (Invitrogen), and then stained for CD45-AF532 (Invitrogen, 51-0451-82, clone 30-F11), Gr-1-PE (BioLegend, 108408, clone RB6-8C5), and F4/80-BV421 (BioLegend, 123137, clone BM8) to delineate hematopoietic lineages, neutrophils, and macrophages. Labeled BALF cells were acquired using a Cytek Aurora flow cytometer and quantified using FlowJo.

### RNA isolation and qRT-PCR.

RNA was isolated from the left lung using the RNeasy Kit (Qiagen). Reverse transcription generated cDNA using iScript (Bio-Rad). Amplification used validated primers (Supplemental Table 10) in a 384-well plate with SsoAdvanced Universal SYBR Green qPCR Supermix (Bio-Rad) in a C1000 Touch Thermocycler (Bio-Rad). Data analysis was performed using the ddCT method in Microsoft Excel and plotted using GraphPad Prism.

### ELISA.

The total protein lysate was prepared from left lung tissue. The KC ELISA kit (R&D Systems) was used according to the manufacturer’s instructions.

### Cell culture and in vitro IR.

Primary mouse PMVECs were isolated from a wild-type C57BL/6J male mouse and cultured in complete EC medium (R&D Systems). Passages 3–9 were used for experiments. Cells were cultured to 80% confluency and then starved in basal medium with 0.05% FBS for 6 hours prior to 10 Gy IR. PBS or TPOm (250 ng/mL) was added to the media 2 hours before IR. After IR, starvation medium was replaced with complete medium with the same supplementation of PBS or TPOm.

### Immunofluorescence staining of cells.

Mouse PMVECs were cultured onto chamber slides and briefly fixed with 4% paraformaldehyde. The slides were incubated with primary antibodies for c-MPL (IBL America, bs-11311R-Biotin) and CD31 (Cell Signaling, 77699T, clone D8V9E), stained with secondary antibodies of Steptavidin-FITC (Thermo Fisher Scientific, SA10002) and anti-rabbit IgG Alexa Fluor 594 (Thermo Fisher Scientific, A-11012), and mounted with VECTASHIELD with DAPI (Vector Laboratories, H-1200-10). Images of cells were captured on a Zeiss LSM 800 Airyscan microscope.

### Western blotting.

Lung tissue lysate was prepared at harvest using RIPA buffer (Thermo Fisher Scientific) with a protease-phosphatase inhibitor cocktail (Thermo Fisher Scientific). For in vitro experiments, cells were plated on 6-well plates and then lysed for protein at 48 hours after IR using RIPA buffer. Protein concentration was determined using Protein assay reagent (Bio-Rad). Proteins were separated by SDS-PAGE, transferred to PVDF membranes, and assessed by blotting with primary antibodies. Tissue Western blots targeted c-MPL (IBL-America, bs-11311R), p21 (Cell Signaling, 64016), p16 (Cell Signaling, 29271, clone E5F3Y), MMP-9 (Cell Signaling, 24317, clone E7N3Y), TIMP-1 (Invitrogen, MA1-773, clone F31 P2 A5), and CD31 (Cell Signaling, 77699T, clone D8V9E). Cell Western blots targeted c-MPL (IBL-America, bs-11311R) and ICAM-1 (Invitrogen, MA5407, clone 1A29). All blots were incubated with horseradish peroxidase–conjugated secondary antibodies (Thermo Fisher Scientific) and detected by using enhanced chemiluminescence (Thermo Fisher Scientific). Densitometry was performed using ImageJ (NIH).

### Neutrophil adhesion assay.

Primary bone marrow (BM) was isolated as described previously (74). Briefly, wild-type C57BL/6J mouse femurs were collected and flushed with PBS to harvest the BM cells. Red blood cells were lysed using ammonium chloride, and BM cells were density gradient separated using histopaque-1119 and histopaque-1077 (Sigma-Aldrich). Isolated neutrophils were labeled with calcein-AM (Thermo Fisher Scientific), as previously described (75), followed by incubating with 10 Gy–irradiated mouse PMVECs treated with PBS or TPOm at 250 ng/mL on 24-well plates for 30 minutes. Wells were washed and immediately imaged with an AMG EVOS fl Digital Inverted microscope (Thermo Fisher Scientific) using the GFP fluorescence channel at ×100 magnification. Wells were also analyzed for total fluorescence intensity at an excitation of 485 nm and emission of 520 nm with SpectraMAX spectrophotometer (Molecular Devices).

### Crystal violet assay.

Mouse PMVECs in 96-well plates were treated with TPOm and IR and then stained with a 0.1% crystal violet solution (Alfa Aesar) for 20 minutes. After washing and drying overnight, wells were imaged with a AMG EVOS fl Digital Inverted microscope (Thermo Fisher Scientific). Crystal violet cell stain was eluted with 100% methanol and analyzed for optical density at 570 nm with SpectraMAX spectrophotometer (Molecular Devices).

### μCT.

For μCT imaging, mice were anesthetized by continuous isoflurane. Imaging was performed on an Inveon Multimodality scanner (Siemens) in which CT x-rays were generated by an 80 kV peak voltage, 0.5 mA current, and 200-millisecond exposure time. The CT field of view was 5.5 cm by 8.5 cm with a 50 μm overall resolution. Analysis was performed using MIM Software. CT images were visually inspected using a 3D projection display to examine for interpretability and image artifact. Manual regions of interest were defined by annotating lungs in the 3D projection, from which lung volume and density were obtained.

### Electrocardiography and respiratory rate.

During μCT imaging, anesthetized mice were connected to a model 1030 ERT module (Small Animal Instruments) via electrodes on their front and left rear paws. Heart rate was calculated by averaging the time between R waves in the electrocardiogram over the range of 25 beats per mouse. Mice were placed prone on top of a respiration pillow sensor (Small Animal Instruments) to collect breath rate data averaged over 2 minutes during CT acquisition.

### Isolation of lung cells for scRNA-Seq.

Lungs from naive, vehicle-, and TPOm-treated mice (2 weeks after WTI) were minced and incubated with collagenase A (2.5 mg/mL; Sigma-Aldrich), Dispase II (1.0 mg/mL; Sigma-Aldrich), and DNase (80 U/mL; Sigma-Aldrich) at 37°C for 30 minutes. Cells were filtered through a 40 μm filter and pooled (*n* = 3 per group) prior to staining. Single cells were stained with CD45-AF532 (Invitrogen, 51-0451-82, clone 30-F11), Hoechst-33342 (Invitrogen), and 7-AAD (BioLegend). Hoechst^+^7-AAD^–^CD45^–^ were isolated as nonhematopoietic lung cells and sorted with a Bigfoot (Thermo Fisher Scientific).

### scRNA-Seq and analysis.

Over 200,000 sorted cells (Hoechst^+^7-AAD^–^CD45^–^) per group were given to Singulomics Corporation for RNA library preparation using Chromium Controller (10X Genomics) and Chromium 3′ Single Cell mRNA-Seq V5 reagents. Libraries were sequenced using an Illumina NextSeq500 sequencer. Raw data were processed using Cell Ranger package (10X Genomics) and analyzed using Seurat v5.0 (76). Naive and vehicle- and TPOm-treated datasets were integrated using the canonical correlation analysis–based integration to remove batch effects. Quality control and filtering removed ribosomal and mitochondrial RNA (>5%) using Seurat package. Subsequent analyses of principle component analysis, UMAP, and clustering were performed. Seurat FindAllMarkers function was used on normalized and scaled data to identify clusters based on top 10 gene expression and canonical gene expression. Differential expression of TPOm versus vehicle in CapECs was assessed using Seurat FindMarkers function with MAST statistical test generating *P* values. Data visualization used tidyverse R packages (77).

### Analysis of enriched biological pathways in scRNA-Seq data.

For gene ontology (GO) pathway analysis, >0.25 log_2_fc up- and downregulated genes were extracted from differential expression analysis. Extracted genes were compared with a *Mus musculus* GO database and organized into enriched biological pathways using the enrichGO function within clusterProfiler package in R (78). *P* values indicated pathway gene set enrichment within expressed genes of the cluster.

### Statistics.

Analyses were performed using GraphPad Prism software version 10. In vivo data are shown as mean ± SEM. In vitro data are shown as mean ± SD. Statistical differences of the three-group (naive, vehicle-, and TPOm-treated) datasets were analyzed using nonparametric methods, using a 1-way ANOVA with Tukey’s test as post hoc comparison. Survival data were analyzed using Kaplan-Meier analysis. A threshold of *P* < 0.05 was considered significant. Differential expression analysis in the scRNA-Seq data used the MAST statistical framework (79).

### Study approval.

Animal studies were approved by the Albert Einstein College of Medicine Institute for Animal Care and Use Committee under protocol 00001154.

### Data availability.

Values for all data points in graphs are reported in the Supporting Data Values file. The scRNA-Seq data generated for this study are deposited in NCBI’s GEO (accession GSE273235). The supporting analytic code for the scRNA-seq analysis can be accessed at https://github.com/guhalab-code/JCI_Insight_Thrombopoietin_mimetic_lung_WTI (branch: main; commit ID: 3948551065629eca2d1fffd5613ab8f97fa2e388).

## Author contributions

JE, GE, WLY, and CG developed the conceptual framework of the study. CG provided overall supervision and project administration. JE designed and performed experiments. JE and SD acquired data. WK performed CT and cardiac and lung function measurements. KET interpreted the histopathology. JE, WLY, and CG analyzed data. JE and WLY drafted the manuscript. JE and WLY edited the manuscript.

## Supplementary Material

Supplemental data

Unedited blot and gel images

Supplemental tables 1- and 4

Supplemental table 10

Supporting data values

## Version 1 11/08/2024

Electronic publication
