# Supplementary material for: Thrombopoietin mimetic reduces mouse lung inflammation and fibrosis after radiation by attenuating activated endothelial phenotypes
Source: JCI Insight. 2024 Nov 8;9(21):e181330. doi: 10.1172/jci.insight.181330 (PMC11601560; doi:10.1172/jci.insight.181330)
Supplement: Supplemental data [file jciinsight-9-181330-s216.pdf]

A

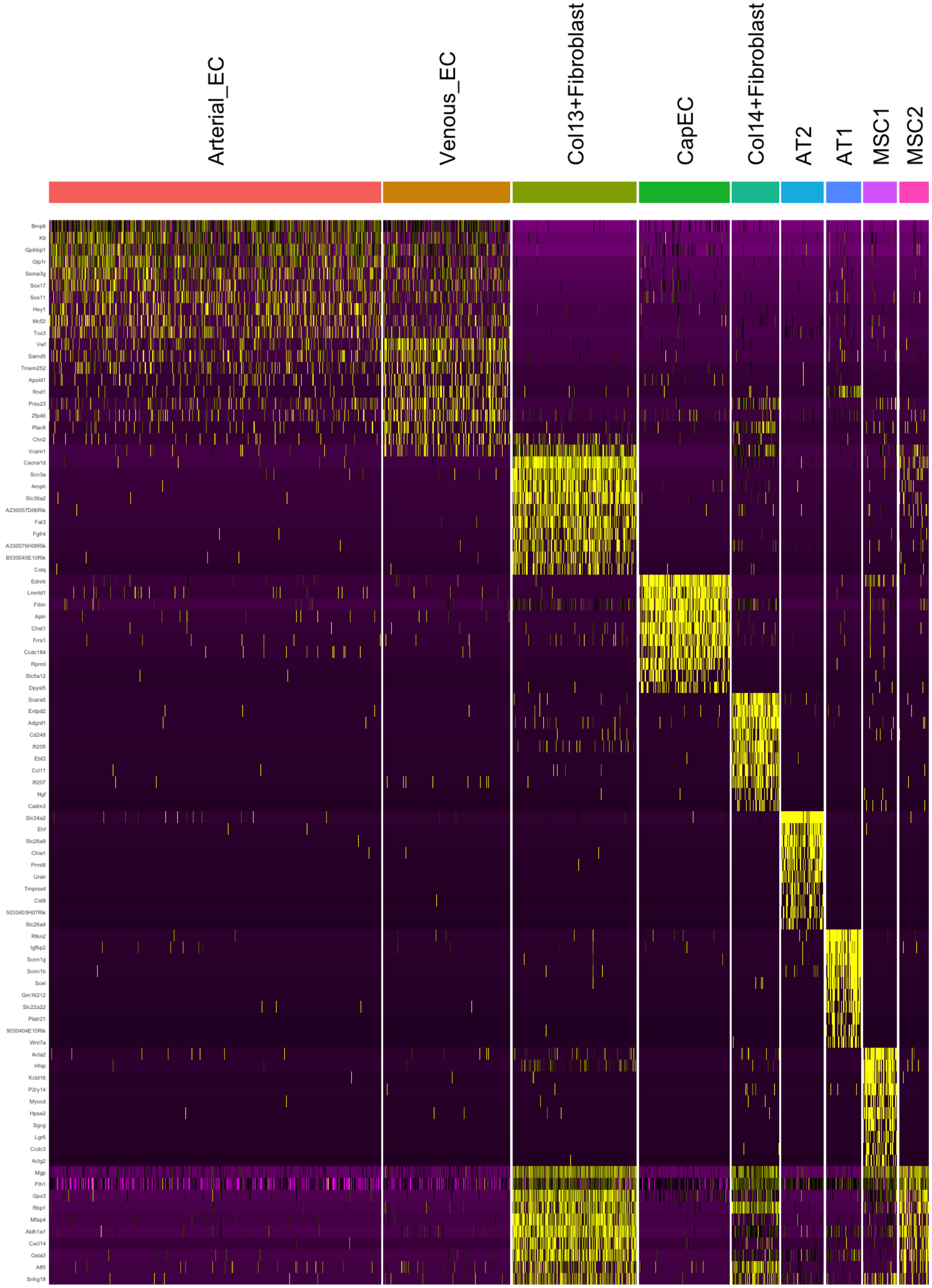
Arterial_EC
Venous_EC
Col13+Fibroblast
CapEC
Col14+Fibroblast
AT2
AT1
MSC1
MSC2
Bmp6
Kit
Gpihbp1
Glp1r
Sema3g
Sox17
Sox11
Hey1
Mcf2l
Tox3
Vwf
Samd5
Tmem252
Apold1
Rnd1
Prss23
Zfp46
Plac8
Chn2
Vcam1
Cacna1d
Scn3a
Amph
Slc36a2
A230057D06Rik
Fat3
Fgfr4
A330076H08Rik
B530045E10Rik
Colq
Ednrb
Lmntd1
Fibin
Apln
Chst1
Frrs1
Ccdc184
Rprml
Slc5a12
Dpysl5
Scara5
Entpd2
Adgrd1
Cd248
Ifi205
Ebf2
Ccl11
Ifi207
Ngf
Cadm3
Slc34a2
Ehf
Slc26a9
Chia1
Prmt8
Urah
Tmprss4
Cst8
5033403H07Rik
Slc26a4
Rtkn2
Igfbp2
Scnn1g
Scnn1b
Scel
Gm16212
Slc22a22
Platr21
9030404E10Rik
Wnt7a
Acta2
Hhip
Kctd16
P2ry14
Myocd
Hpse2
Sgcg
Lgr6
Ccdc3
Actg2
Mgp
Fth1
Gpx3
Rbp1
Mfap4
Aldh1a1
Cxcl14
Gsta3
Atf5
Snhg18

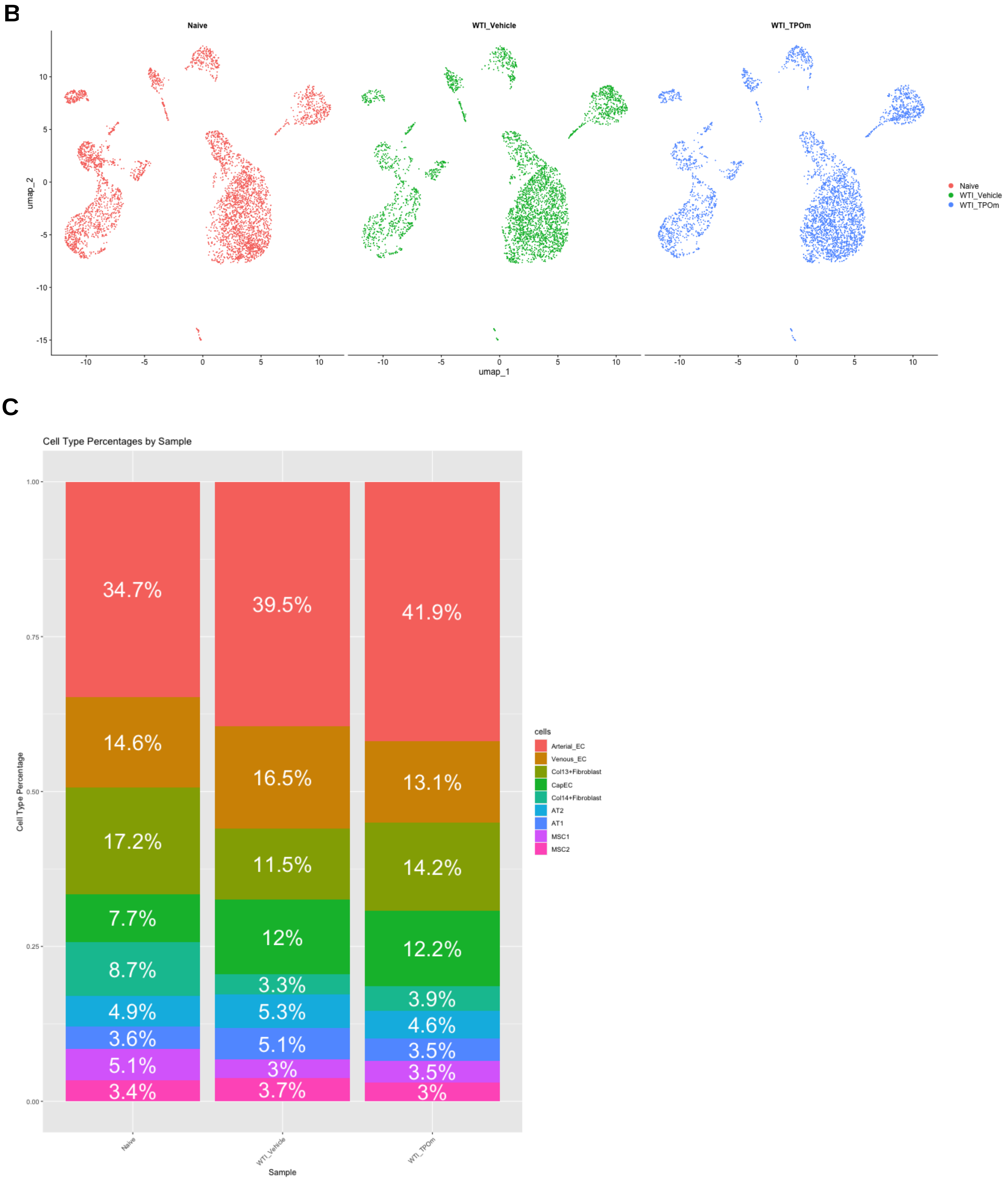

Supplemental Figure 1. Overview of clusters in lung cells analyzed by scRNA-Seq and their distributions in each treatment group. (**A**) Heatmap of the most differentially expressed genes of lung cells in the 9 different clusters. The color bars indicate gene expression level in log2 scale. (**B**) Two-dimensional UMAP projection split by treatment group. 4451 cells in naïve. 3925 cells in vehicle. 3862 cells in TPOm. (**C**) Each cluster's distribution by percentage iterated by treatment condition.

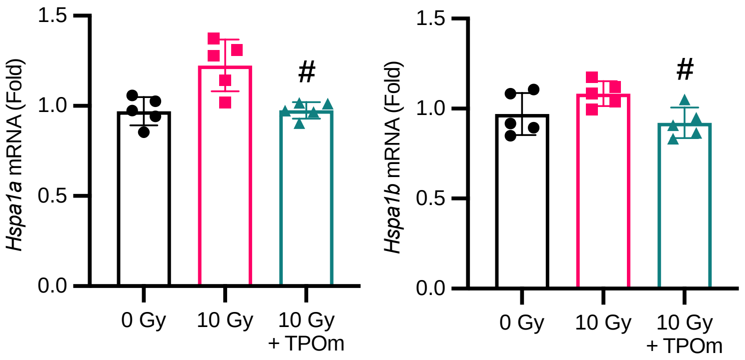

Supplemental Figure 2. The mRNA expression of *Hspa1a* and *Hspa1b* in mouse PMVECs. Mouse primary PMVECs were isolated from C57B/6J mouse and cultured. PMVECs from 0 Gy control and irradiated at 10 Gy 4 days after IR. The irradiated MPVECs were pretreated with PBS and TPOm 2 hours before IR. The mRNA expression of *Hspa1a* and *Hspa1b* was determined by qPCR. The results of qPCR analysis are normalized with *Hprt1* as an internal control and are expressed as fold change compared with the naive group. Data are shown as means ± SD (n=5/group). *P < 0.05 *vs.* 0 Gy and $^{\#}P$ < 0.05 *vs.* 10 Gy. Data were analyzed using non-parametric methods, using a one-way ANOVA with Tukey test as post-hoc comparison.
