# Supplementary material for: Thrombopoietin mimetic reduces mouse lung inflammation and fibrosis after radiation by attenuating activated endothelial phenotypes
Source: JCI Insight. 2024 Nov 8;9(21):e181330. doi: 10.1172/jci.insight.181330 (PMC11601560; doi:10.1172/jci.insight.181330)
Supplement: Unedited blot and gel images [file jciinsight-9-181330-s217.pdf]

Figure 3A

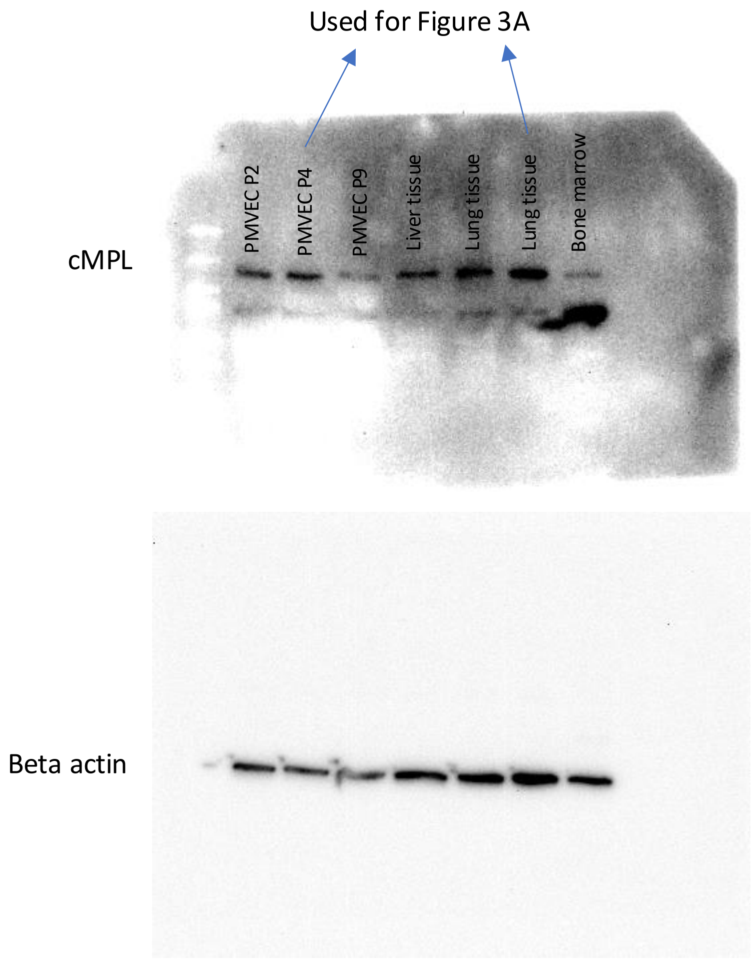

Used for Figure 3A
PMVEC P2
PMVEC P4
PMVEC P9
Liver tissue
Lung tissue
Lung tissue
Bone marrow
cMPL
Beta actin

Figure 3E

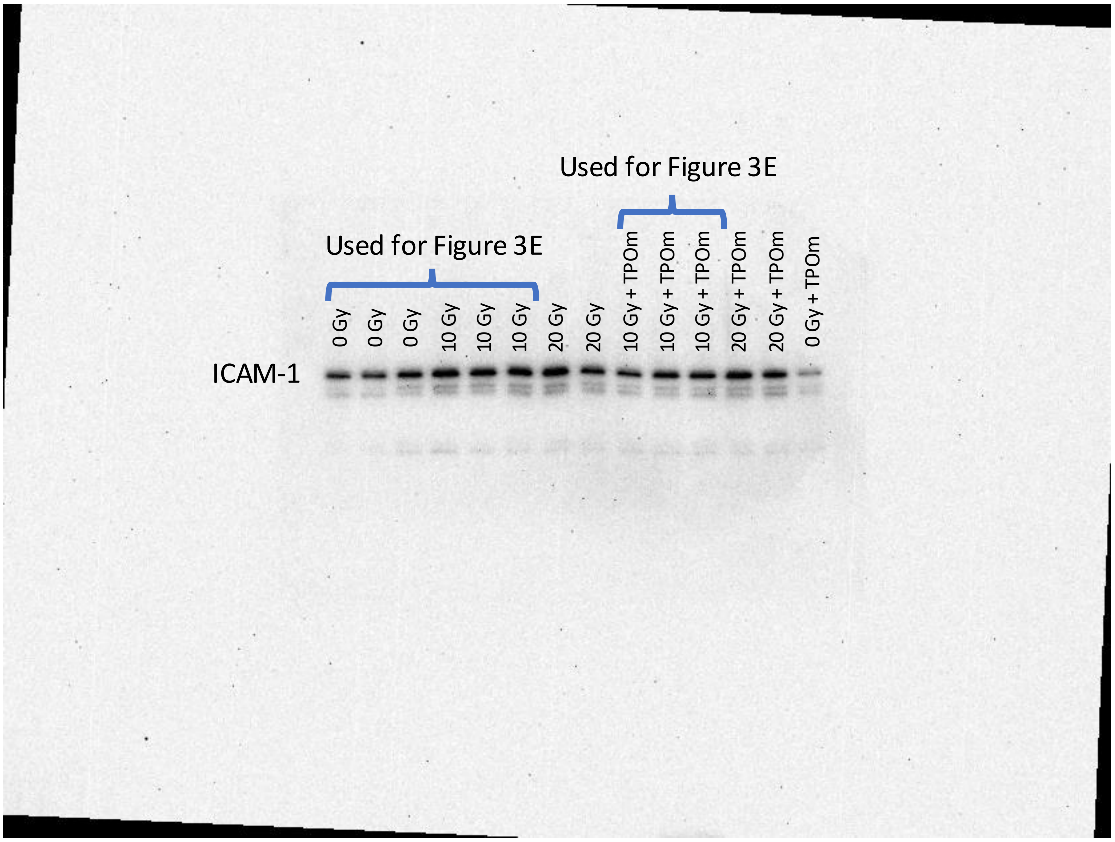

Used for Figure 3E
Used for Figure 3E
0 Gy
0 Gy
0 Gy
10 Gy
10 Gy
10 Gy
20 Gy
20 Gy
10 Gy + TPOm
10 Gy + TPOm
10 Gy + TPOm
20 Gy + TPOm
20 Gy + TPOm
0 Gy + TPOm
ICAM-1

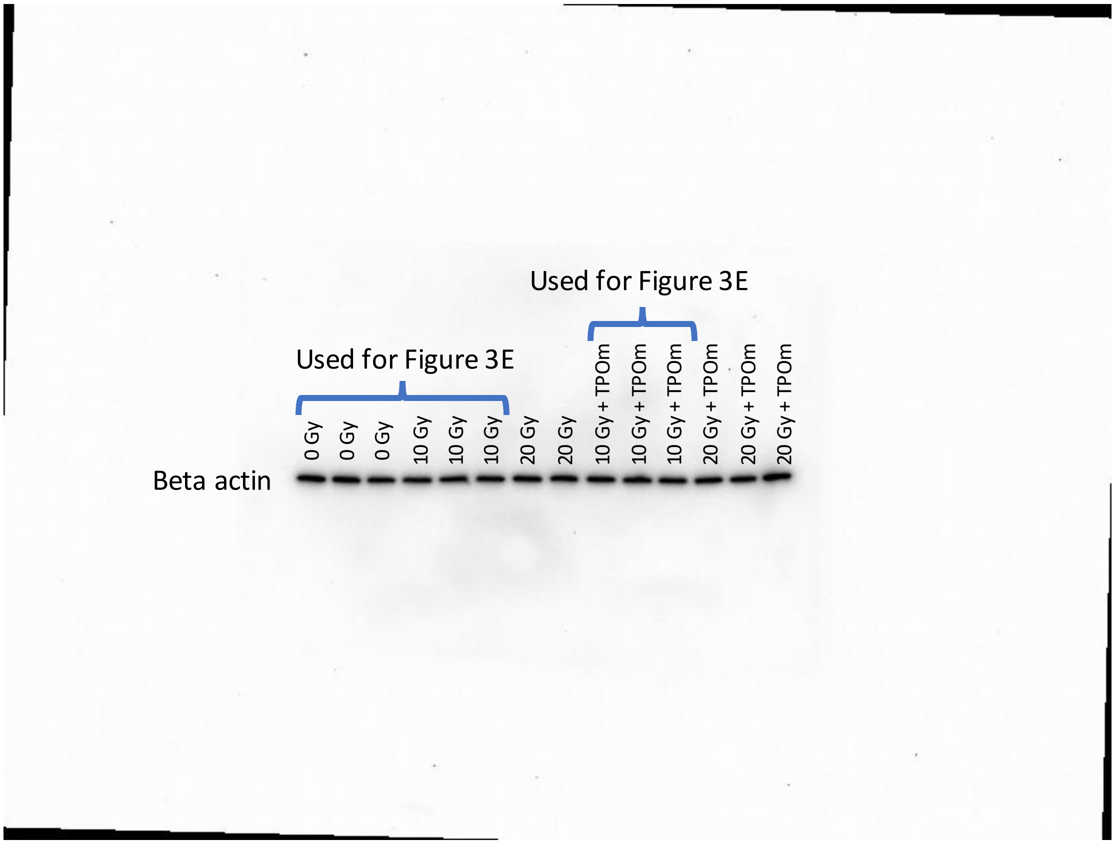

Used for Figure 3E
Used for Figure 3E
0 Gy
0 Gy
0 Gy
10 Gy
10 Gy
10 Gy
20 Gy
20 Gy
10 Gy + TPOm
10 Gy + TPOm
10 Gy + TPOm
20 Gy + TPOm
20 Gy + TPOm
20 Gy + TPOm
Beta actin

Figure 4F

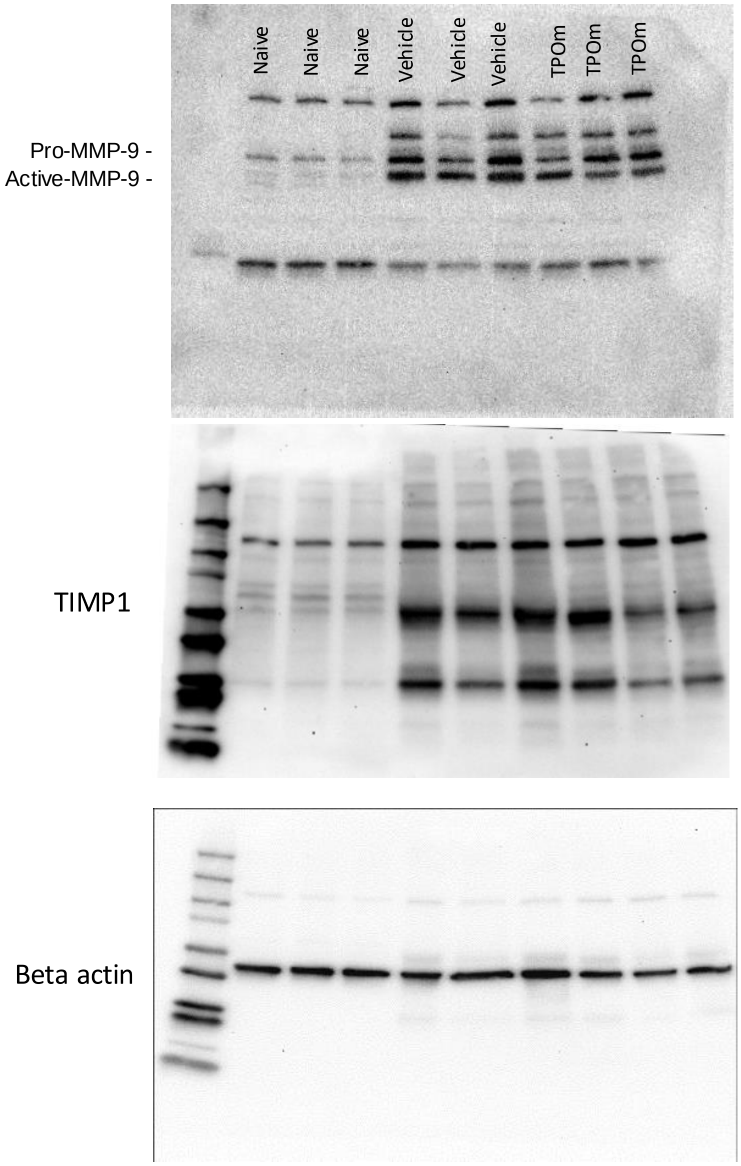
Naive
Naive
Naive
Vehicle
Vehicle
Vehicle
TPOm
TPOm
TPOm
Pro-MMP-9 -
Active-MMP-9 -
TIMP1
Beta actin

Figure 5E

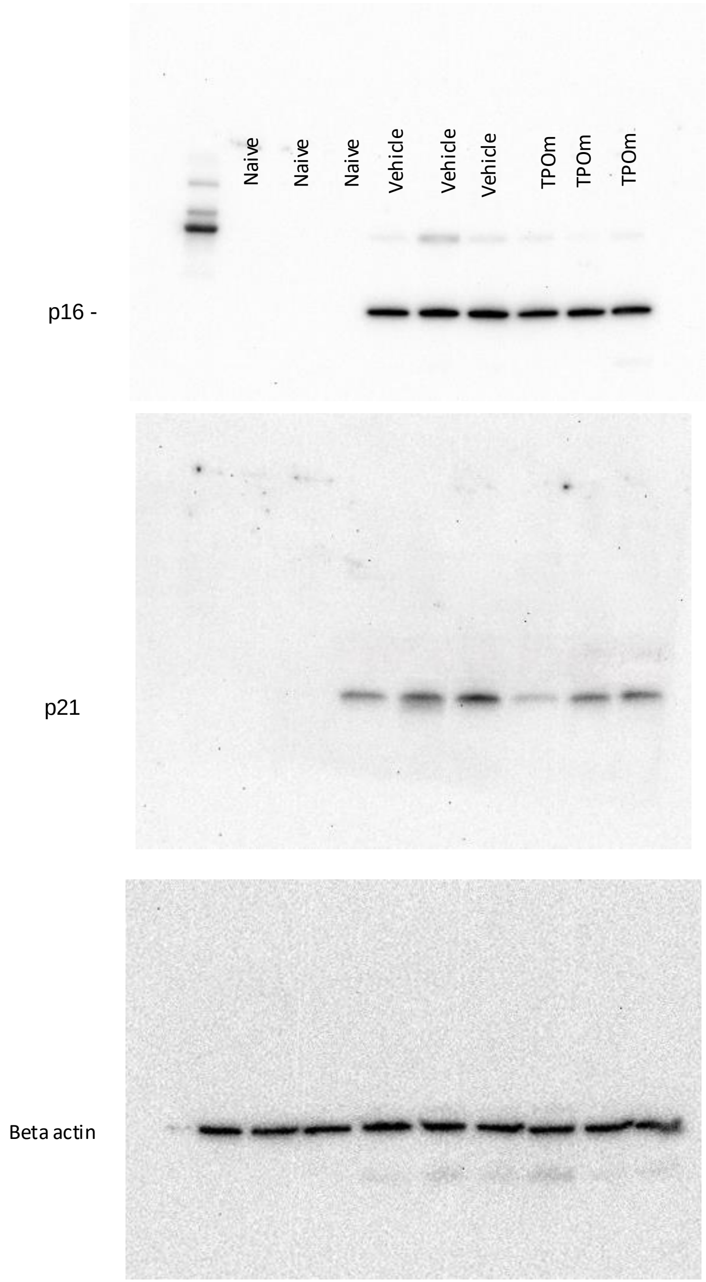

Naive
Naive
Naive
Vehicle
Vehicle
Vehicle
TPOm
TPOm
TPOm
p16 -
p21
Beta actin

Figure 6B

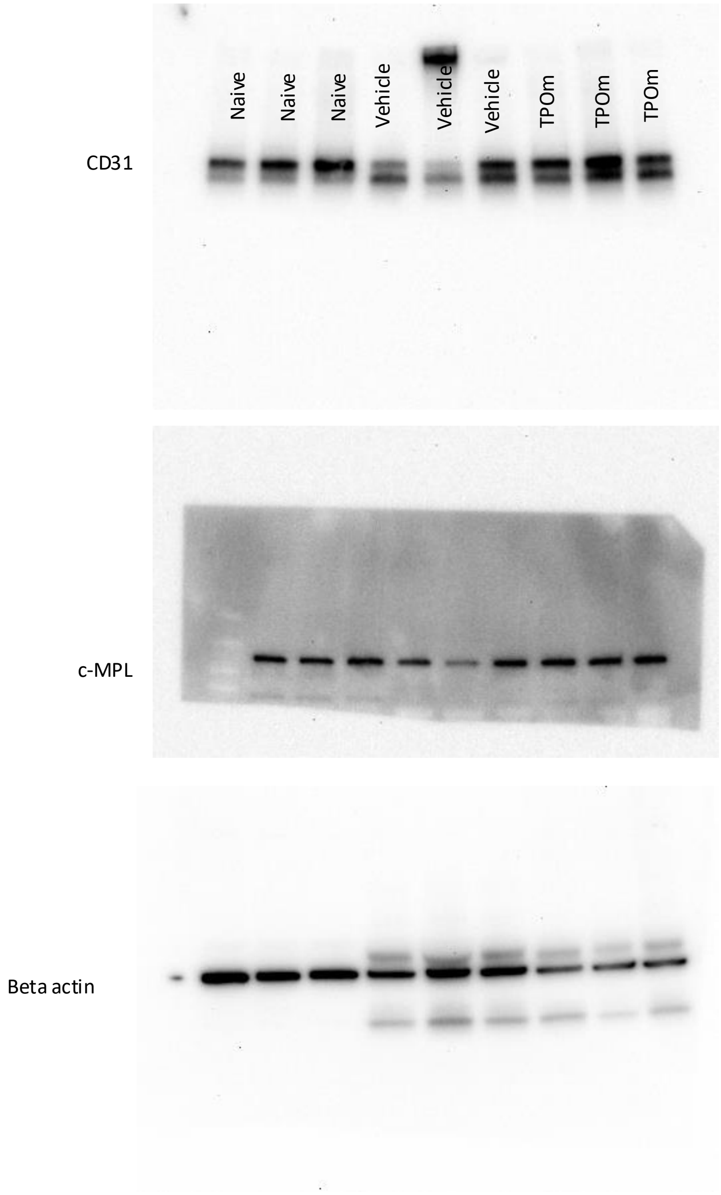
Naive
Naive
Naive
Vehicle
Vehicle
Vehicle
TPOm
TPOm
TPOm
CD31
c-MPL
Beta actin

Figure 8J

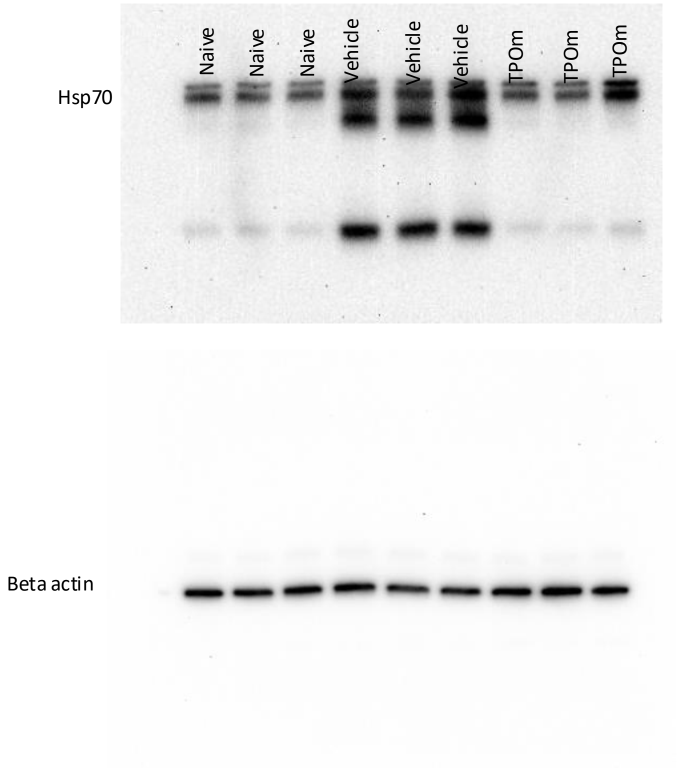
Naive
Naive
Naive
Vehicle
Vehicle
Vehicle
TPOm
TPOm
TPOm
Hsp70
Beta actin
